# A double-blind, 377-subject randomized study identifies *Ruminococcus, Coprococcus, Christensenella*, and *Collinsella* as long-term potential key players in the modulation of the gut microbiome of lactose intolerant individuals by galacto-oligosaccharides

**DOI:** 10.1080/19490976.2021.1957536

**Published:** 2021-08-07

**Authors:** M. A. Azcarate-Peril, J. Roach, A. Marsh, William D. Chey, William J. Sandborn, Andrew J. Ritter, Dennis A. Savaiano, T. R. Klaenhammer

**Affiliations:** a Department of Medicine, Division of Gastroenterology and Hepatology, School of Medicine, University of North Carolina, Chapel Hill, NC, USA; b UNC Microbiome Core, Center for Gastrointestinal Biology and Disease, School of Medicine, University of North Carolina, Chapel Hill, NC, USA; c UNC Information Technology Services and Research Computing, University of North Carolina, Chapel Hill, NC, USA; d Departments of Internal Medicine and Nutritional Sciences, University of Michigan Health System, Ann Arbor, MI, USA; e Division of Gastroenterology, University of California San Diego, La Jolla, CA, USA; f Ritter Pharmaceuticals, Inc, Los Angeles, CA, USA; g Department of Nutrition Science, Purdue University, West Lafayette, IN, USA; h Department of Food, Bioprocessing and Nutrition Sciences, North Carolina State University, Raleigh, NC, USA

**Keywords:** Prebiotics, microbiome modulation, lactose intolerance, human lactase, *Bifidobacterium*, short chain fatty acids, galacto-oligosaccharides, GOS

## Abstract

**Background**. Our recent publication (Chey et al., Nutrients 2020) showed that a 30-day administration of pure galacto-oligosaccharides (GOS) significantly reduced symptoms and altered the fecal microbiome in patients with lactose intolerance (LI). **Results**. In this addendum, we performed an in-depth analysis of the fecal microbiome of the 377 LI patients randomized to one of two GOS doses (Low, 10–15 grams/day or High, 15–20 grams/day), or placebo in a multi-center, double-blinded, placebo-controlled trial. Sequencing of 16S rRNA amplicons was done on GOS or placebo groups at weeks zero (baseline), four (end of treatment), nine, 16 and 22. Taxa impacted by treatment and subsequent dairy consumption included lactose-fermenting species of *Bifidobacterium, Lactobacillus, Lactococcus*, and *Streptococcus*. Increased secondary fermentation microorganisms included *Coprococcus* and *Ruminococcus* species, *Blautia producta*, and *Methanobrevibacterium*. Finally, tertiary fermenters that use acetate to generate butyrate were also increased, including *Faecalibacterium prausnitzii, Roseburia faecis*, and *C. eutactus*. **Conclusions**. Results confirmed and expanded data on GOS microbiome modulation in LI individuals. Microbiome analysis at 16 and 22 weeks after treatment further suggested relatively long-term benefits when individuals continued consumption of dairy products.

## Introduction

Expression of the human intestinal lactase-phlorizin hydrolase (lactase) naturally declines as we age.^1^ Although most human individuals cannot digest lactose in high quantities, most can tolerate approximately one cup of milk a day, whereas individuals of Northern European descent can consume high amounts of lactose-containing foods over their lifetime with no adverse effects.^2,3^ Individuals that show specific symptoms (diarrhea, flatulence, bloating and others) upon consumption of low amounts of lactose are considered lactose intolerant.

The first human gut microbiome genome wide association study (mGWASs) to identify human genes and pathways correlated with the microbial composition was performed using data generated by the Human Microbiome Project (HMP) in 2015.^4^ The study showed that persistent expression of the lactase gene (*LCT*) in the small intestine, correlated with *Bifidobacterium* abundance and the rs2164210 Single Nucleotide Polymorphism (SNP) in *LCT*.^4,5^ Historic data have demonstrated that consumption of prebiotics, specifically galacto-oligosaccharides (GOS), result in increased abundance of lactose-fermenting *Bifidobacterium* (a phenomenon termed “bifidogenic effect”).^6–9^ The clinical and associated microbiome studies with GOS provide a clear rationale for the use of prebiotics to increase tolerance to lactose in affected individuals through enhancement of the lactose-fermenting microbes, most notably *Bifidobacterium* populations.

Previous studies showed that lactose-intolerant individuals treated with a high-purity prebiotic galacto-oligosaccharide (GOS), with a subsequent diet that included dairy products, exhibited a clinical response toward lactose tolerance^10^ and a shift of the gut microbiome.^11^ In a randomized, double-blind, parallel group, placebo-controlled study conducted at two sites in the United States, responder data for abdominal pain (subjects who reported over a 50% decrease in abdominal pain from baseline) showed that 72% of subjects receiving GOS responded to treatment compared to 28% in the placebo group. Additionally, 50% of patients in the treatment group who reported abdominal pain at the beginning of the study reported no abdominal pain after GOS treatment and 30-days after re-introducing dairy (compared with 17% in the placebo group).^10^ Study of the microbiome of the lactose intolerant subjects treated with GOS showed increased lactose-fermenting *Bifidobacterium, Faecalibacterium*, and *Lactobacillus*. Additionally, increased abundance of bifidobacteria correlated with a reduction in abdominal pain and cramping.^11^

The parent study to this addendum,^12^ a clinical data in a multi-center, double-blinded, placebo-controlled trial, showed that 40% of the individuals in the GOS groups had a ≥ 4-point reduction or no symptoms in the LI composite score compared to 26% with placebo (*p* = .043). Additionally, GOS treatment led to significantly higher levels of milk and dairy intake and significant improvements in global assessments compared to placebo. In this addendum, we present the analysis of the fecal microbiome of 377 patients with lactose intolerance (LI), randomized to one of two doses of GOS (GOS High or GOS Low doses), or placebo.

## Results

### Experimental design and analysis approach

Our recently published study, of which this article is an addendum, included 15 investigative centers throughout the U.S. and 3 phases: a screening phase, a treatment phase and a post-treatment phase.^12^ There was a 7-day screening phase where patients were assessed for LI symptoms based on a hydrogen breath test and a blinded-lactose challenge. Stool samples were collected (baseline). Patients were then stratified into Placebo (powdered corn syrup), Low GOS (10–15 grams/day) and High GOS treatments (15–20 grams/day) and administered treatment for 30 days, during which patients did not consume lactose (week 4). Following treatment for 30 days, a stool sample was collected. Then, “real-world” dairy intake was encouraged without further treatment, LI symptoms were assessed, and stool samples were collected after a 30-day period (week 9). Finally, an extension study monitored a subset of subjects (n = 100) for approximately 6 months (week 16) and 12 months post-treatment (week 22).

### High GOS had a marginal impact on diversity of the gut microbiome

**Figure 1.**
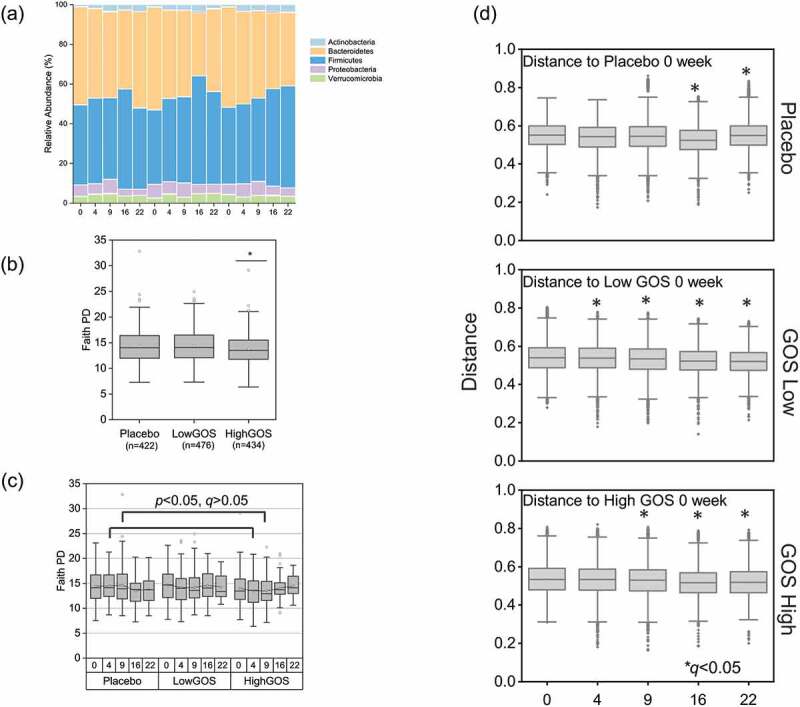
(a) Phylum composition of the gut microbiome of individuals that received either placebo or GOS treatments (Low or High GOS). The most abundant taxa are indicated , . (b) Faith Phylogenetic Diversity (PD) averages between treatments and (c) between treatments at different times. (d) Pairwise PERMANOVA comparisons between Unifrac Unweighted distances by treatment and times. *Corrected *p (q)<*0.05

Sequencing of 16S rRNA amplicons targeting the variable region 4 of the ribosomal gene was performed on samples corresponding to treatment groups: (1) Placebo, (2) GOS Low, (3) GOS High (N = 1,332). Amplicon sequencing yielded a total of 254,386,079 sequences (188,994 mean reads per sample). Overall, sequencing data assigned the majority of Operational Taxonomic Units (OTUs) to the phyla *Firmicutes* averaging 38.9 ± 22.4% per treatment group and time, *Bacteroidetes* (36.5 ± 18.2%), *Proteobacteria* (4.1 ± 1.3%), *Verrucomicrobia* (3.3% ± 2%), and *Actinobacteria* (2.3% ± 1.7%). The *Archaea Euryarchaeota* and the phylum *Tenericutes* were represented at 0.21 ± 0.24, and 0.13 ± 0.11, respectively. The phyla *Cyanobacteria, Fusobacteria, OD1, Synergistetes, TM7, Lentisphaerae, Elusimicrobia, Spirochetes* were identified in a range from 0.003% to 0.07%, and the phyla [*Thermi], Chlorobi, Deferribacteres, Planctomycetes, Armatimonadetes, SR1, Crenarchaeota* (*Archaea*), and *Aquificae* had the lowest representation (from 7.02 × 10^−7^ to 2.4 × 10^−5^%) (Figure 1a).

Analysis of combined data (values from all weeks grouped by treatment) showed overall marginal but significant differences (Kruskal Wallis *p* < .05) in Faith Phylogenetic Diversity (PD) index values between treatments (Figure 1). The difference was driven by the High GOS treatment group, which had a significantly lower diversity. Pairwise comparisons showed that, specifically, the High GOS treatment group had lower diversity values compared to placebo when comparisons were made by week, at weeks 4 and 9, although the differences were not significant after correction for multiple comparisons (Kruskal Wallis with Benjamini-Hochberg adjusted pairwise comparisons *q* > 0.05). No significant differences were observed between placebo and the combined GOS treatments (data not shown).

Permutational multivariate analysis of variance (PERMANOVA) using unweighted Unifrac distance matrices showed statistically significant correlations (Pseudo-F > 2, *p* = .05) between microbiome composition and treatment, as well as treatment over time (Figure 1c, d). Our analysis showed significant differences associated with treatment over time when we compared each time point with the same group at time zero weeks (*p* < .05, *q* < 0.05). Differences between treatments at week 4 were observed for Low GOS (*p* < .05). To confirm the observed differences, we determined how beta diversity changed across time within and between treatment groups using q2-longitudinal.^13^ We visualized individual trajectories in volatility plots using first distances. A linear mixed-effects (LME) test indicated a significant impact from visit number (*P* = .004) on baseline Jaccard distance (Figure S1).

### Differential analysis of taxa impacted by treatment and time

**Figure 2.**
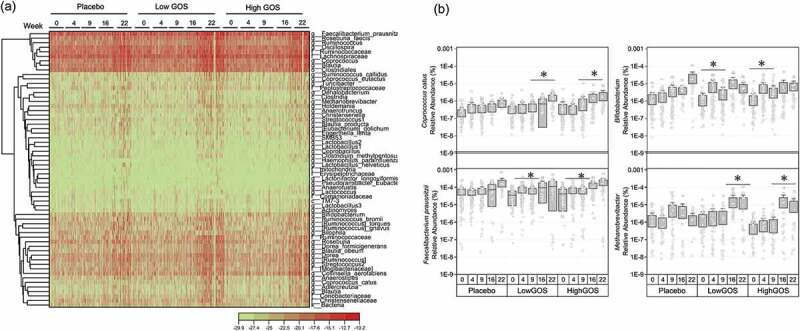
(a) Representation of taxa at genus level differentially represented in at least one group and one time point (FDR corrected Kruskal-Wallis *P* <.05). The heatmap was generated using log2-transformed data in the Heat Map with Dendrogram app within OriginPro 9.7.5.184. (b) Relative abundance by treatment of *Coprococcus catus, Bifidobacterium, Faecalibacterium prausnitzii* and *Methanobrevibacterium* over time (in weeks). *Kruskall-Wallis FDR-corrected *p* < .05

OFrom a total of 605 species-level taxa identified by ANCOM analysis,^14^ 61 were differentially represented in response to treatments by visit week (either at 4, 9, 16 or 22 weeks) after treatment (Figure 2a). The differentially represented groups were distributed among the phyla *Firmicutes* (48 species-level taxa), *Actinobacteria* (6), *Proteobacteria* (4), TM7 (1), *Euryarchaeota* (an *Archaeae*, 1), and one uncharacterized Bacteria. Of a total of 48 Firmicutes species impacted by treatments over time, 38 were of the order *Clostridiales*, four were of the order *Erysipelotrichales*, seven of the *Lactobacillales*, and one of the *Turicibacterales*. Within *Clostridiales*, two families (*Lachnospiraceae* and *Ruminococcaceae*) were the most affected by treatments and showed an increasing linear trend. Important butyrate-producers including *Coprococcus catus*, within the *Lachnospiraceae*,^15^ and *Faecalibacterium prausnitzii*, within the *Ruminococcaceae*, showed an overall significantly increased abundance associated with both low and high GOS treatment groups (Figure 2b). *Lactobacillus, Lactococcus*, and *Streptococcus* species showed a marked increase in relative abundance in response to GOS. Finally, an uncharacterized species of the family *Christensenellaceae* had markedly higher relative abundance in response to GOS during the follow-up weeks, suggesting long-term effects of treatment.

Of the *Actinobacteria, Bifidobacterium* showed a specific abundance increase in response to the treatments (Figure 2b). *Egerthella lenta* and *Collinsella aerofaciens* showed long-term increasing trends, which could be related to increased dairy consumption. Likewise, *Adlercreutzia, Actinomyces*, and an uncharacterized group of the family *Coriobacteriaceae* showed specific increases in abundance associated with the GOS treatments at the follow-up visits (weeks 16 and 22) suggesting long-term effects of the prebiotic treatment in combination with increased consumption of dairy foods. This was also observed in the relative abundance over time of *Methanobrevibacterium*, a methane-producing, commensal *Archaeae* of the healthy microbiome (Figure 2b).

### *Impact of treatment on* Bifidobacterium *and* Lactobacillus *species determined by quantitative (q) PCR*

**Figure 3.**
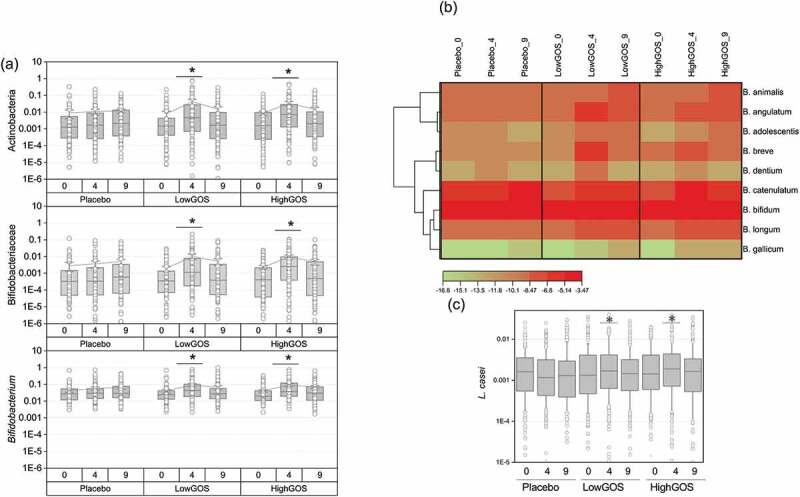
(a) Abundance of the phylum *Actinobacteria*, family *Bifidobacteriaceae*, and genus *Bifidobacterium* determined by high-throughput (HT) qPCR by treatment and time at weeks 0 (baseline), 4 (end of GOS treatment), and 9 (end of trial, lactose challenge). (b) Heat map showing abundance of *Bifidobacterium* species by treatment and time. (c) Abundance of *Lactobacillus casei* determined by HT qPCR. *p < .05

A total of 1050 samples corresponding to 345 subjects receiving either placebo or GOS treatments were analyzed by high-throughput qPCR^16^ using specific 16S rRNA gene, IS and GroEL probes to determine abundance of bifidobacteria and lactobacilli. Confirming our previous observations,^11^ the data showed significant increases in the relative abundance of the phylum Actinobacteria, the family *Bifidobacteriaceae*, and the genus *Bifidobacterium* in response to treatment in both GOS Low and GOS High groups, but not in the placebo group (Figure 3a). At week 9, the abundance of these taxa returned to baseline (week 0) levels. Nine relevant species of *Bifidobacterium* (*B. longum, B. gallicum, B. dentium, B. catenulatum, B. breve, B. bifidum, B. animalis, B. angulatum* and *B. adolescentis*) were also quantified (Figure 3b). Our data showed that overall, the most abundant *Bifidobacterium* species were *B. bifidum* and *B. catenulatum*, both of which were enriched by GOS treatments. *B. angulatum, B. longum*, and *B. breve* increased in the treatment groups at week 4, while *B. animalis* increased at week 9.

Our previous study^11^ showed that 90% (27/30) individuals in the treatment group had an increased abundance of bifidobacteria in response to GOS. In this study, the composite scores calculated as the average of HT qPCR values for all bifidobacteria taxa for each participant showed increased bifidobacteria in 72.3% (71/94) participants in the Low GOS group and 72.5% (74/102) in the High GOS group compared to only 46.8% (50/94) in the placebo. Further analysis of responders versus non-responders presented in Table S1 shows percent of responders (measured as a positive value when subtracting abundance at day 31 or day 61 from baseline) versus non-responders at days 31 and 61. Individuals that had a non-detectable abundance of the taxon at time 0 were not included in the analysis. The phylum *Actinobacteria*, the family *Bifidobacteriaceae* and genus *Bifidobacterium* were increased at 4 weeks in the GOS treatments with 73.7%, 77.8%, and 65.7% subjects showing a positive response, respectively. Likewise, *B. angulatum, B. gallicum* and *B. longum* were increased only at 4 weeks in both treatments. *B. bifidum, B. breve*, and *B. catenulatum* were increased at days 31 and 61 suggesting a long-term effect of the prebiotic treatment.

Taxonomic groups relevant to genus *Lactobacillus* were quantified, including *Firmicutes, Lactobacillaceae, L. acidophilus, L. casei, L. crispatus, L. delbrueckii, L. gasseri, L. murinus, L. reuteri*, and *L. rhamnosus*. Of the analyzed taxa, differences reached statistical significance for *L. casei* (Figure 3c).

### GOS effects associated with patient characteristics

**Figure 4.**
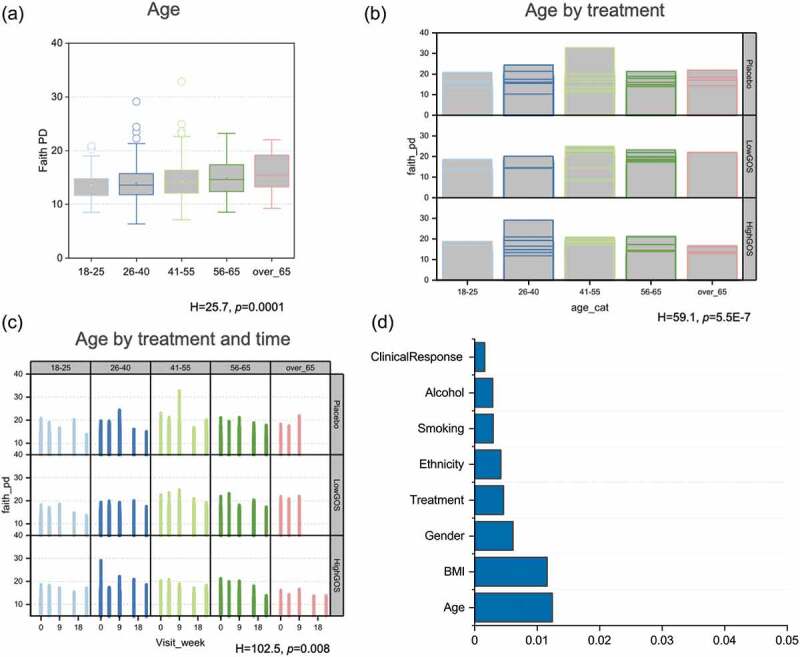
Analysis of alpha diversity (Faith Phylogenetic Diversity values) by (a) age, (b) age by treatment, and (c) age by treatment and time. H and *p* values from Kruskall-Wallis analysis indicating the impact significance of the factor considered on diversity are denoted the bottom of each figure. (d) Adonis (PERMANOVA) R^2^ values representing effect size of patients’ characteristics and treatment on microbiome composition

Faith phylogenetic diversity (Faith PD) and Shannon diversity indices showed that age (Figure 4 and Supplementary material) significantly impacted baseline diversity of the cohort, increasing over time. Combined analysis of the population characteristic with treatment and with treatment by time showed that treatment had different effects depending on baseline diversity and specific category (Figure S2). Of the analyzed characteristics, age and BMI had the larger effect size (Figure 4d). PERMANOVA using unweighted and weighted Unifrac distance matrices showed statistically significant correlations (Pseudo-F > 1, *p* = .05) between microbiome composition and age, gender, BMI, race, ethnicity, alcohol consumption and smoking (Table S2). These categories were still significant after combining with treatment and treatment plus visit number.

### Predicted functional differences between treatment groups

**Figure 5.**
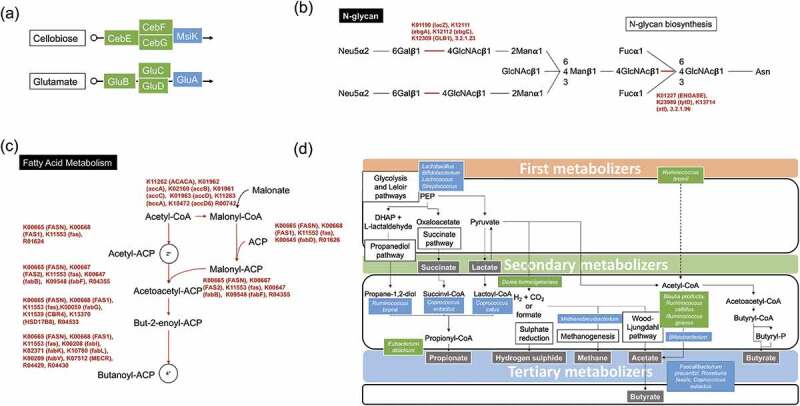
Predicted functionality of the GOS-enhanced microbiota. (a) The cellobiose and glutamate bacterial transporters were overrepresented in the prebiotic group. (b) Pathway KO00511 (Other glycan degradation), with genes overrepresented in the GOS group in the N-glycan biosynthesis pathway. (c) Genes responsible for initiation of fatty acid biosynthesis overrepresented in the GOS groups. (d) Summary of the gut metabolic processes and potentially responsible microorganisms that participate in biotransformation of GOS. Blue boxes indicate organisms known to carry out the enzymatic process, while green boxes are potential new players in the intestinal cross feeding of GOS. Depiction was based on our results and published research studies.^17–21^

Phylogenetic Investigation of Communities by Reconstruction of Unobserved States (PICRUSt) analysis was applied to 16S rRNA amplicon sequencing data to assess potential differences in microbial functional capabilities between groups. Group significance analysis on metagenome predictions identified 58 KEGG pathways differentially represented between placebo and GOS groups combined at 4 weeks. Of those, 34 pathways were significantly (Mann-Whitney test, FDR corrected *p* <.05) overrepresented in the GOS group, and 24 were overrepresented in the placebo group. Overall, the prebiotic group had increased representation of carbohydrate metabolism pathways (galactose, pentose and glucuronate interconversion, glycolysis/gluconeogenesis), lipid metabolism (fatty acid biosynthesis, butyrate), and amino acid metabolism (phenylalanine, tryptophan, cysteine and methionine). Relative abundance of six bacterial transport genes was increased in the GOS group. Abundance of the gene responsible for fermentation of the prebiotic (β-galactosidase, EC 3.2.1.23) was increased in the GOS groups. This enzyme is also represented in the pathway KO00511 (Other glycan degradation), in the N-glycan biosynthesis (Figure 5b).

Genes responsible for initiation of fatty acid biosynthesis were overrepresented in the GOS groups (Figure 5c). These included FabH (3-ketoacyl- acyl carrier protein [ACP] synthase III), which catalyzes the condensation of acetyl-CoA with malonyl-ACP to yield acetoacetyl-ACP and has transacylase activity, transferring the acetate moiety from actyl-CoA to acetyl-ACP. Acetyl-ACP is then condensed with malonyl-ACP by FabB (synthase I) or by FabF (synthase II), also overrepresented in the GOS groups. The pathway for initiation of fatty acid biosynthesis, which involves decarboxylation of malonyl-ACP by FabH, FabB or FabF to form acetyl-ACP followed by subsequent condensation with malonyl-ACP, was also overrepresented in the GOS groups. All the enzymatic steps involved in elongation of fatty acids from Butyryl-ACP to Stearoyl-CoA were overrepresented in the GOS groups. Also of relevance is that the enzymatic step to convert Acetyl-CoA to Malonyl-CoA is present in the metabolism of propionic acid, a carboxylic short chain fatty acid (SCFA). Likewise, this step is important in the biosynthesis of fatty acids. Furthermore, the type I Fatty acid synthase gene (Fas), essential for subsequent steps in the biosynthetic pathway, was overrepresented in the GOS groups.

Based on previous and new data from this study, we attempted to summarize how GOS are metabolized by the gut bacterial network. We included species that were differentially represented in the GOS treatment groups and classified them as first metabolizers or degraders (*Bifidobacterium, Lactobacillus, Streptococcus, Ruminococcus bromii*), secondary metabolizers, which use the intermediate metabolites (including lactate and succinate) for generation of SCFAs, hydrogen sulfide and methane, and tertiary metabolizers that use a SCFA (acetate) to generate another (butyrate).

### Supervised learning analysis

Supervised learning was used in this study to build a descriptive model of the data to identify a highly predictive subset of taxa for further investigation. Significantly increased abundance of *Bifidobacterium* (Other), *B. adolescentis* and *B. pseudolongum*, and decreased abundance of the families *Lachnospiraceae* and *Christensenellaceae*, and the genera *Roseburia, Ruminococcus, Coprobacillus*, and *Eubacterium dolichum* characterized the GOS-treated groups (High and Low GOS combined). Data suggest that GOS primarily acts on lactose intolerant individuals by increasing the abundance of rapid lactose metabolizers (e.g. bifidobacteria). *Bifidobacterium* spp. generate lactate and acetate, which can then be used by other species to generate butyrate.

## Discussion

Galacto-oligosaccharides (GOS) are known to modulate the gut microbiome, increasing the abundance, and enhancing functionality of beneficial bacteria.^11,22–24^ An initial trial and subsequent microbiome analysis of lactose-intolerant individuals^10,11^ showed that patients receiving a high purity GOS preparation exhibited decreased symptoms. This decrease in symptoms was correlated with increases in bifidobacteria, lactobacilli and faecalibacteria. In this addendum to the second clinical study on lactose intolerant individuals^12^ and in accordance with our first microbiome report,^11^ most of the groups impacted by treatment or subsequent dairy consumption, in addition to bifidobacteria, were of the phylum *Firmicutes*. The genera *Lactobacillus, Lactococcus*, and *Streptococcus*, all lactate-producers, showed a specific response to GOS treatments at week 4. Our study added potential new players to the cross-feeding intestinal network. For example, *R. bromii*, over represented in the GOS groups, has been shown to be an essential species in the degradation of resistant starch in the human colon.^17^ A more recent *in vitro* study further established the ability of the species to metabolize resistant starch and confirmed the cross feeding between species where *Ruminococcus gnavus*, which prefers mucin to grow, can use the products of starch degradation generated by *R. bromii*.*^18^* Although the study of Crost et al.^18^ did not specifically test GOS, it included glucose oligosaccharides, and led us to speculate that *R. bromii* could use GOS, thereby generating intermediate products that can result in the production of SCFAs. Additional work is needed to investigate this possibility.

In our study, important commensal groups within the *Actinobacteria* (*Egerthella lenta, Collinsella aerofaciens, Adlercreutzia, Actinomyces*, and an uncharacterized group of the *Coriobacteriaceae* family) and the *Firmicutes* (an uncharacterized species of the family *Christensenellaceae*) as well as the *Archaea Methanobrevibacterium* showed a markedly higher relative abundance in response to GOS; notably when subjects continued consumption of dairy products. *C. aerofaciens* is a common gut commensal that breaks down di- and oligosaccharides generating acetate, lactate, formate, and H_2_^25,26^ , with at least one strain producing butyric acid.^27^ This is in accordance with a recently published study showing that bifidobacteria, though proficient at degrading resistant starch and inulin, may not be the most important contributor to the specific butyrogenic effects of fermentable fibers in the short term.^19^ Nevertheless, bifidobacteria do catabolize prebiotic fibers (both GOS and FOS) and generate lactate and acetate that can fuel secondary degraders which produce other SCFAs.

*Coprococcus catus* was one major species that showed an overall significant increased abundance in correlation with treatment only. Our previous study did not specifically identify *C. catus* as increased by the treatment or subsequent period of dairy consumption;^11^ however, an Operational Taxonomic Unit (OTU) identified at the time at the family level only (Lachnospiraceae_2) was increased in response to GOS and dairy consumption. The genus *Coprococcus* (family *Lachnospiraceae*, phylum *Firmicutes*) contains three species (*C. eutactus, C. catus* and *C. comes*), which are not phylogenetically closely related.^28^ *C. catus* produces butyrate and propionate, while *C. eutactus* and *C. comes* produce butyrate with formate or lactate, respectively. *C. catus* uses lactate to generate propionate via the acrylate pathway.^29^ The role of propionate in intestinal and overall health was only recently elucidated, with studies showing that propionate can lower serum cholesterol levels, lipogenesis, and carcinogenesis risk.^30^ Propionate also promotes secretion of the satiety-inducing hormones PYY and GLP-1 hormones in human colonic cells.^31–34^

Given the variable abundance of *Bifidobacterium* in the gut of human adults,^6,11,23^ not all subjects in our study demonstrated a bifidogenic response; however, 77.8% of subjects showed a *Bifidobacteriaceae* increase. Of the *Bifidobacterium* species quantified by HT qPCR, *B. bifidum, B. catenulatum*, and *B. longum* were increased in >60% of subjects. Although a previous study reported that *B. bifidum* was most found in infants and *B. catenulatum* in adult individuals,^35^ in our study both species represented the most abundant gut bifidobacteria overall.

Predictive analysis of functionality showed that the prebiotic groups had increased representation of carbohydrate metabolism pathways (galactose, pentose and glucuronate interconversion, glycolysis/gluconeogenesis) suggesting an increased saccharolytic potential. It is generally accepted that bacteria driven largely by saccharolytic metabolism (*i.e*., no proteolytic activity) are potentially beneficial.^36–38^ Conversely, the placebo group overrepresented genes were involved in the degradation of the amino acids valine, leucine, and isoleucine, as well as in the metabolism of cysteine and methionine.^39^ An overrepresentation of genes encoding cellobiose and glutamate transporters was observed in the GOS groups. Three genes corresponded to the cellobiose transport system and three genes to the transport of glutamate (Figure 5a). These observations suggest that the cellobiose transport system might be used for GOS transport into bacterial cells. Cellobiose, a disaccharide comprised two β-glucose molecules linked by a β(1→4) glycosidic linkage formed by glucose and galactose, is structurally like lactose, and transporters for this carbohydrate have been extensively characterized in Lactic Acid Bacteria (LAB), including *Lactobacillus* and *Bifidobacterium* species.^40,41^ As previously reported,^11^ GOS increased the abundance of the genes responsible for GOS transport^42^ as well as catabolism of lactose (β-galactosidase, EC 3.2.1.23).

Finally, genes responsible for initiation of fatty acid biosynthesis and all the enzymatic steps involved in elongation of fatty acids from Butyryl-ACP and from there, ultimately, to Stearoyl-CoA, were overrepresented in the GOS groups. Saturated long-chain fatty acid (SLCFA)-producing bacteria have been recently shown to contribute to regulation of the gastrointestinal motility in rats.^43^ In this study, excess intracolonic SLCFAs were associated with increased motility in a rodent model of neonatal maternal separation, and correlated with increased abundance of *Prevotella, Lactobacillus, Alistipes*, and *Ruminiclostridium*. Bacterial fatty acid biosynthesis pathways have also been targeted for antibiotic discovery.^44^ In this sense, the ability to manipulate these pathways without the use of antibiotics could be an attractive therapeutic approach to infections. The results indicate not only a modification of the composition of the gut microbiome, but a clear restructuring of the microbiome functionality.

Observations from this study are novel and add to the knowledge of how to approach treatment and ameliorate symptoms for lactose intolerance, while identifying new potential long-term key bacterial players and networks in the metabolism of prebiotics in the gastrointestinal tract.

## Conclusion

Correcting imbalances in the gut microbial communities that can be correlated with disease is one of the main goals of microbiome research. This study confirms and expands on the modulation of beneficial and commensal bacteria by GOS in individuals clinically diagnosed as lactose intolerant, thus advancing the notion that gut microbial disproportions can be adjusted to improve quality of life. Beyond the traditional, potentially probiotic bacteria (*Bifidobacterium, Lactobacillus, Faecalibacterium*), we have identified new bacterial taxa (*Ruminococcus, Coprococcus, Christensenella, Collinsella*) that may be a part of the beneficial gut network enhanced by treatment, with the potential consequence of long term increased saccharolytic potential and, hence, the ability to consume dairy products. Further research is needed to predict treatment response and therefore advance personalized disease management.

## Materials and methods

The lower dose of GOS (RP-G28) was 5 grams twice daily on days 1–10 followed by 7.5 grams twice daily on days 11–30. The higher dose of GOS (RP-G28) was 7.5 g twice daily for days 1–10, followed by 10 g twice daily on days 11–30. The placebo group received a powdered corn syrup that matched the consistency, color, sweetness, and taste of the drug. This clinical trial can be found on the clinical trial registry website (www.clinicaltrials.gov), trial number NCT02673749.

### DNA isolation

Stool samples (200 mg) were transferred to sterile 2 ml tubes containing 200 mg of glass beads, ≤11 μm (Sigma, St. Louis, MO) and 1.4 ml of Qiagen ASL buffer (Valencia, CA). Bead-beating was then carried in a Qiagen TissueLyser II at 30 Hz. Subsequently, samples were incubated at 95ºC for 5 minutes and centrifuged at 21,000 × g for 3 minutes. To remove PCR inhibitors, supernatants were transferred to new 2 ml-tubes containing InhibiEx inhibitor adsorption tablets (Qiagen) and vortexed vigorously. After a brief centrifugation, supernatants were aspirated and transferred to a new tube with Qiagen AL buffer containing Proteinase K (600IU/μl). Samples were then incubated at 70◦C for 10 minutes. DNA was purified using a standard on-column purification method with Qiagen buffers AW1 and AW2 as washing agents and eluted in 10 mM Tris (pH 8.0).

### 16S rRNA amplicon sequencing

12.5 ng of total DNA were amplified using universal primers 515 F-806 R targeting the V4 region of the bacterial 16S rRNA gene.^45,46^ Primer sequences contained overhang adapters appended to the 5ʹ end of each primer for compatibility with Illumina sequencing platform. Master mixes contained 12.5 ng of total DNA, 0.2 µM of each primer and 2x KAPA HiFi HotStart ReadyMix (KAPA Biosystems, Wilmington, MA). Each 16S amplicon was purified using the AMPure XP reagent (Beckman Coulter, Indianapolis, IN). In the next step each sample was amplified using a limited cycle PCR program, adding Illumina sequencing adapters and dual‐index barcodes (index 1(i7) and index 2(i5)) (Illumina, San Diego, CA) to the amplicon target. The final libraries were again purified using the AMPure XP reagent (Beckman Coulter), quantified and normalized prior to pooling. The DNA library pool was then denatured with NaOH, diluted with hybridization buffer and heat denatured before loading on the Illumina HiSeq 2500 instrument. Automated cluster generation and paired–end 2 × 250 bp sequencing with dual reads were performed according to the manufacturer’s instructions.

### Sequencing data analysis

Multiplexed paired end fastq files were produced from the sequencing results of the Illumina HiSeq using the Illumina software configure BclToFastq. The paired-end fastq files were joined into a single multiplexed, single-end fastq using the software tool fastq-join. Demultiplexing and quality filtering were performed on the joined results. Quality analysis reports were produced using the FastQC software. Bioinformatics analysis of bacterial 16S rRNA amplicon sequencing data was conducted using the Quantitative Insights into Microbial Ecology (QIIME) software versions 1 and 2^47,48^ at a 25,000 reads/sample depth. OTU picking was performed on the quality filtered results using pick_de_novo_otus.py in QIIME and using the DADA2 plugin in QIIME 2. Chimeric sequences were detected and removed using ChimeraSlayer. Alpha diversity and beta diversity analysis were performed on the data set using the QIIME routines: alpha_rarefaction.py and beta_diversity_through_plots.py,^49,50^ respectively. Summary reports of taxonomic assignment by sample and all categories were produced using QIIME summarize_taxa_through_plots.py and summarize_otu_by_cat.py. Longitudinal analysis of alpha and beta diversity was performed using the QIIME 2 longitudinal plugin.^13^

### *High-throughput quantitative PCR detection of* Bifidobacterium *and* Lactobacillus *species.*

The access array AA 24.192 (Fluidigm Corporation, San Francisco, CA) validated in previous studies^51–54^ was used for quantification of the following taxonomic groups: domain *Bacteria*, phylum *Actinobacteria*, genus *Bifidobacterium* and *Bifidobacterium* species. The taxonomic groups targeted in the *Lactobacillus* array included: phylum *Firmicutes*, genus *Lactobacillus*, and species of the genus *Lactobacillus*. Pre-amplification (specific target amplification, STA) assays and microfluidic qPCR were performed on a BioMark HD reader as described.^24^ Raw data were normalized using the Livak method.^55^ Cq values for each sample were normalized against their respective Cq value obtained from Universal primers using the equation: Ratio (reference/target) = 2 – ^Ct (ref)-Ct (target)^. One-way ANOVA (ANalysis Of VAriance) with post-hoc Tukey tests were applied for comparing multiple treatments.

### PICRUSt analysis of 16S rRNA amplicon sequencing data

Analysis of 16S rRNA amplicon sequencing data was performed using the default settings of PICRUSt (version 0.9.1). The resulting metagenomic data were submitted to the HMP unified metabolic analysis network (HUMAnN2)^56^ pipeline to sort individual genes into Kyoto Encyclopedia of Genes and Genomes (KEGG) pathways representing varying proportions of each imputed sample metagenome.

### Supervised classification via the random forests classifier using the QIIME script supervised_learning.py

OTUs present in less than 10 samples were filtered out. The OTU table was normalized using DeSeq prior to applying supervised_learning.py in QIIME. We ran 10-fold cross validation on the normalized OTU table to obtain more robust estimates of the generalization error and feature importance (including standard deviations). We then produced a single results file containing the average estimated generalization error of the classified, and the pooled standard deviation. The baseline error for random guessing was 80%.

## Supplementary Material

Supplemental Material

## Data Availability

16S rRNA amplicon sequencing data has been submitted to the Sequence Read Archive (SRA) and is available under the BioProject accession number PRJNA727279.

## Addendum for

Chey, W.; Sandborn, W.; Ritter, A.J.; Foyt, H.; Azcarate-Peril, M.A.; Savaiano, D.A. Galacto-Oligosaccharide RP-G28 Improves Multiple Clinical Outcomes in Lactose-Intolerant Patients. Nutrients 2020, 12, 1058. https://doi.org/10.3390/nu12041058
