# Supplementary material for: A double-blind, 377-subject randomized study identifies Ruminococcus, Coprococcus, Christensenella, and Collinsella as long-term potential key players in the modulation of the gut microbiome of lactose intolerant individuals by galacto-oligosaccharides
Source: Gut Microbes. 2021 Aug 7;13(1):1957536. doi: 10.1080/19490976.2021.1957536 (PMC8354614; doi:10.1080/19490976.2021.1957536)
Supplement: Supplemental Material [file KGMI_A_1957536_SM7339.zip › Supplementary information/Supplementary Material captions.docx]

**Supplementary Material**

**Table S1.** Bifidogenic responders versus non responders in placebo and treatment groups at 31 and 61 days. Treatment groups are: 1: Placebo, 2: GOS – low dose, 3: GOS – high dose.

**Table S2.** Permutational Analysis of Variance (PERMANOVA) analysis using unweighted and weighted Unifrac distance matrices between microbiome composition and single parameters (age, gender, BMI, race, ethnicity, alcohol consumption and smoking), and between microbiome composition and treatment by factor.

**Table S3.** High-throughput qPCR primers targeting bacterial groups and species within the *Lactobacillus* genus using the Access Array AA 24.192 (Fluidigm Corporation, San Francisco, CA, USA).

**Figure S1**. **(a)** Volatility charts of longitudinal change in unweighted UniFrac distances between successive samples collected from the same subject (first distances) over time by treatment, **(b)** Linear mixed-effects model results for Jaccard distances between stool bacterial compositions of subjects by visit number and treatment.

**Figure S2.** Analysis of alpha diversity (Faith Phylogenetic Diversity and Shannon diversity values) by factor (age, BMI, race, ethnicity, gender, clinical response, alcohol consumption and smoking habits, left panel), factor by treatment (middle panel), and factor by treatment and time (right panel). H and *p* values are indicated at the bottom of each figure.

**Figure S3.** Principal Component Analysis (PCA) plots of significantly impacted taxa identified by ANCOM analysis by race, ethnicity, gender, clinical response, alcohol consumption and smoking. Original values were ln(x)-transformed. Unit variance scaling was applied to rows; SVD with imputation was used to calculate principal components. X and Y axis show principal component 1 and principal component 2. Prediction ellipses are such that with probability 0.95, a new observation from the same group will fall inside the ellipse.
