# Supplementary material for: A double-blind, 377-subject randomized study identifies Ruminococcus, Coprococcus, Christensenella, and Collinsella as long-term potential key players in the modulation of the gut microbiome of lactose intolerant individuals by galacto-oligosaccharides
Source: Gut Microbes. 2021 Aug 7;13(1):1957536. doi: 10.1080/19490976.2021.1957536 (PMC8354614; doi:10.1080/19490976.2021.1957536)
Supplement: Supplemental Material [file KGMI_A_1957536_SM7339.zip › Supplementary information/Supplementary_Figures_Addendum.pptx]

## Slide 1
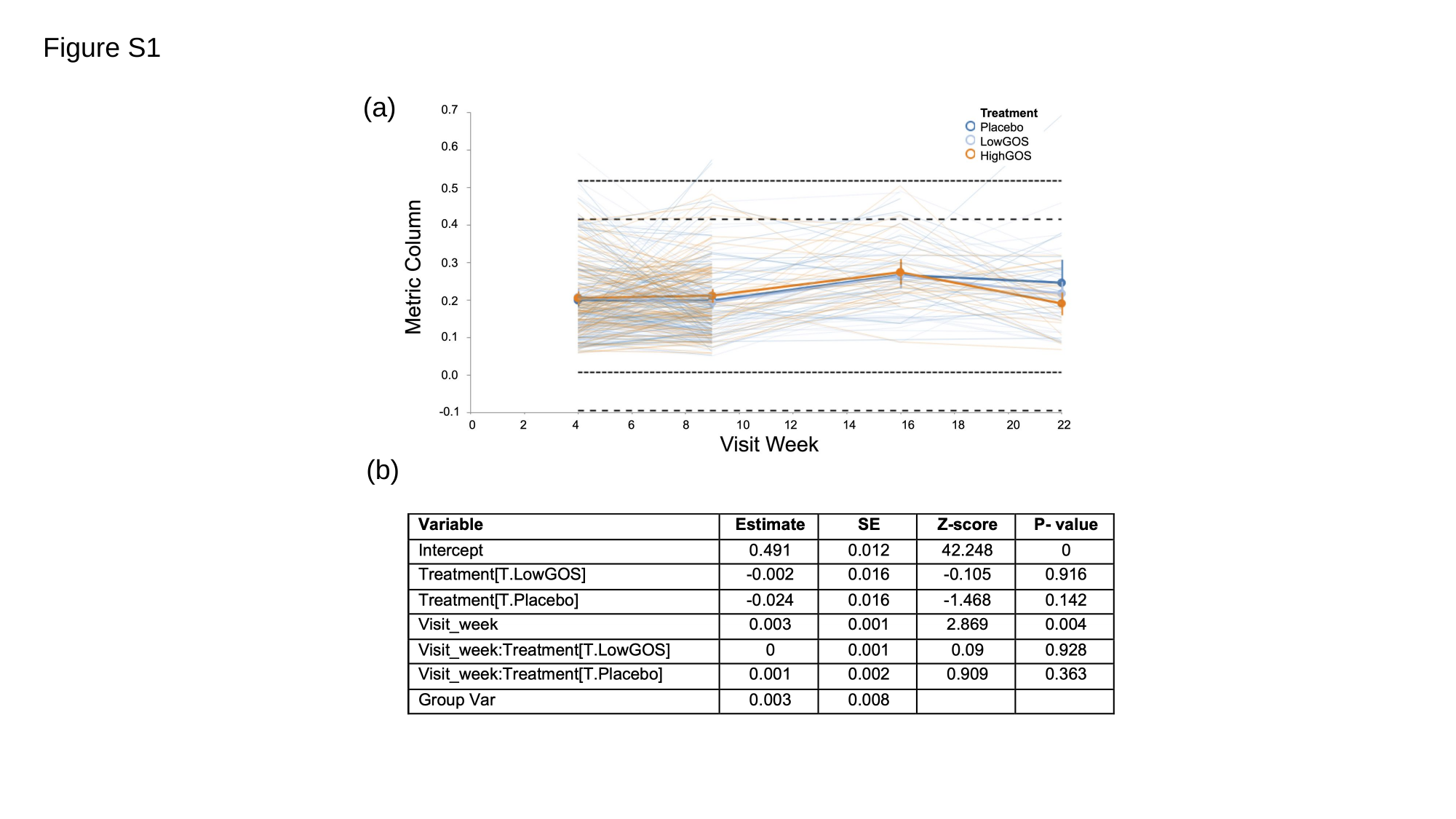

Figure S1
(a)
(b)

## Slide 2
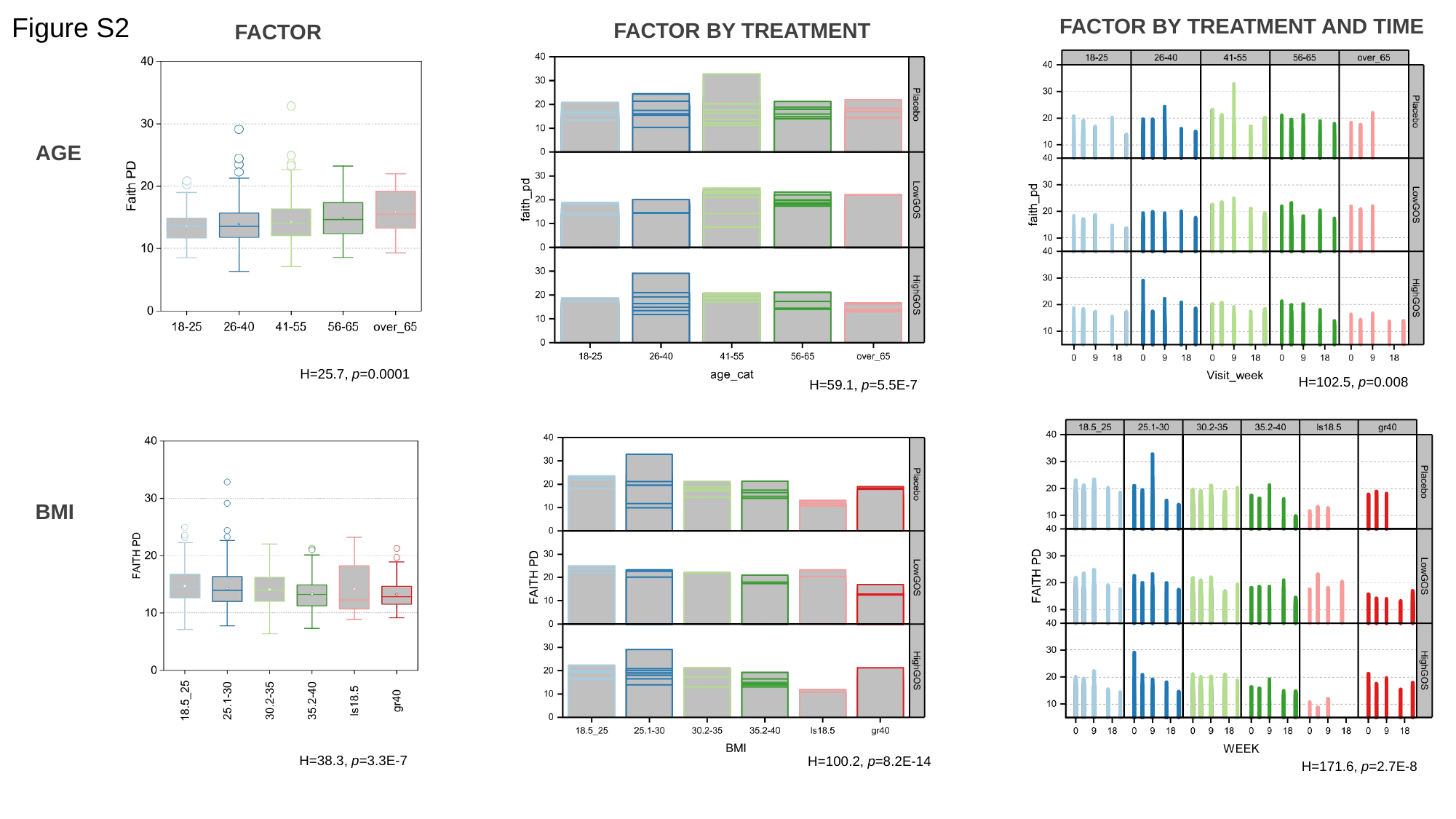

Figure S2
FACTOR BY TREATMENT AND TIME
FACTOR BY TREATMENT
FACTOR
AGE
H=25.7, p=0.0001
H=102.5, p=0.008
H=59.1, p=5.5E-7
BMI
H=38.3, p=3.3E-7
H=100.2, p=8.2E-14
H=171.6, p=2.7E-8

## Slide 3
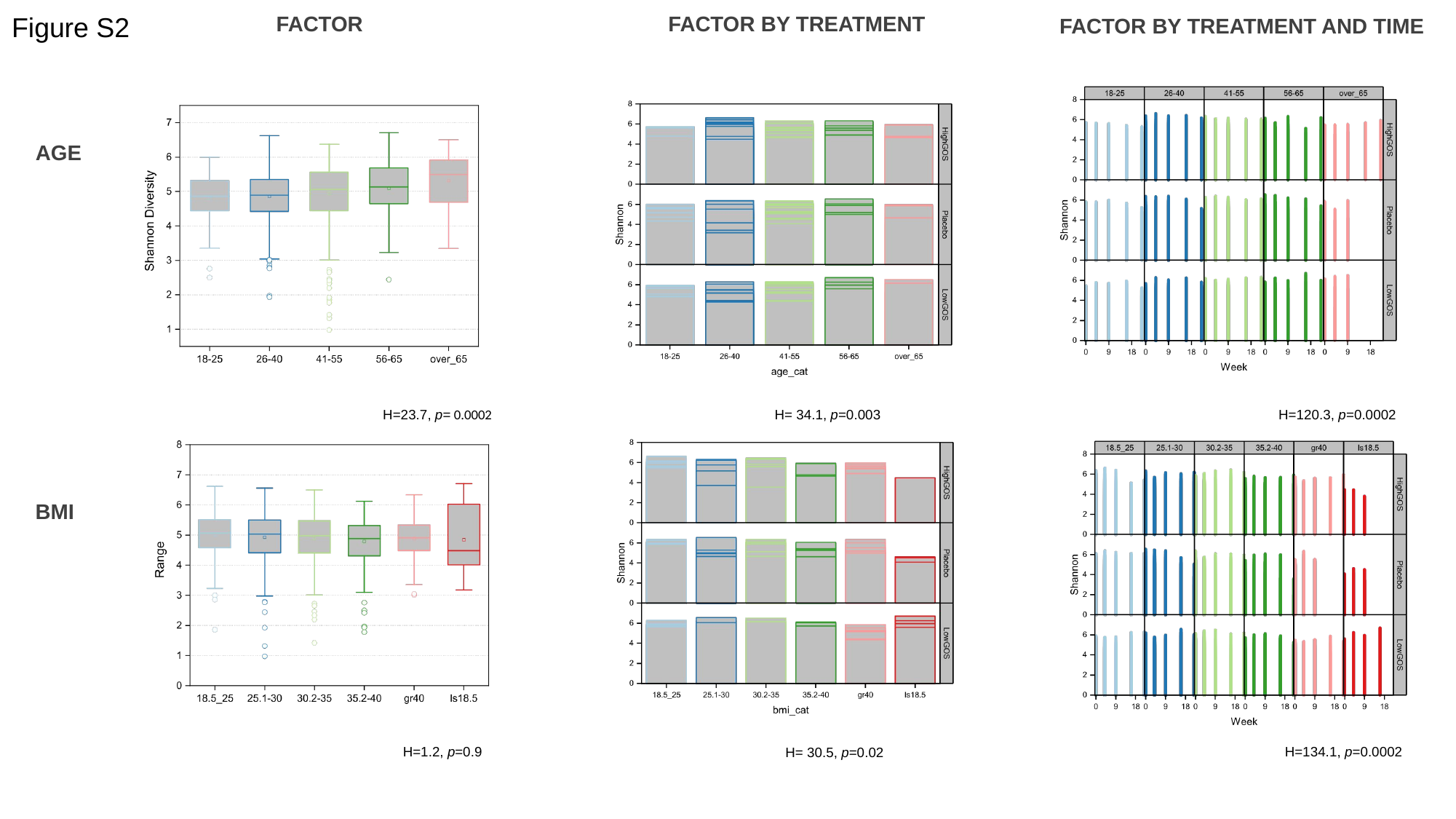

Figure S2
FACTOR
FACTOR BY TREATMENT
FACTOR BY TREATMENT AND TIME
AGE
H=120.3, p=0.0002
H=23.7, p= 0.0002
H= 34.1, p=0.003
BMI
H=1.2, p=0.9
H=134.1, p=0.0002
H= 30.5, p=0.02

## Slide 4
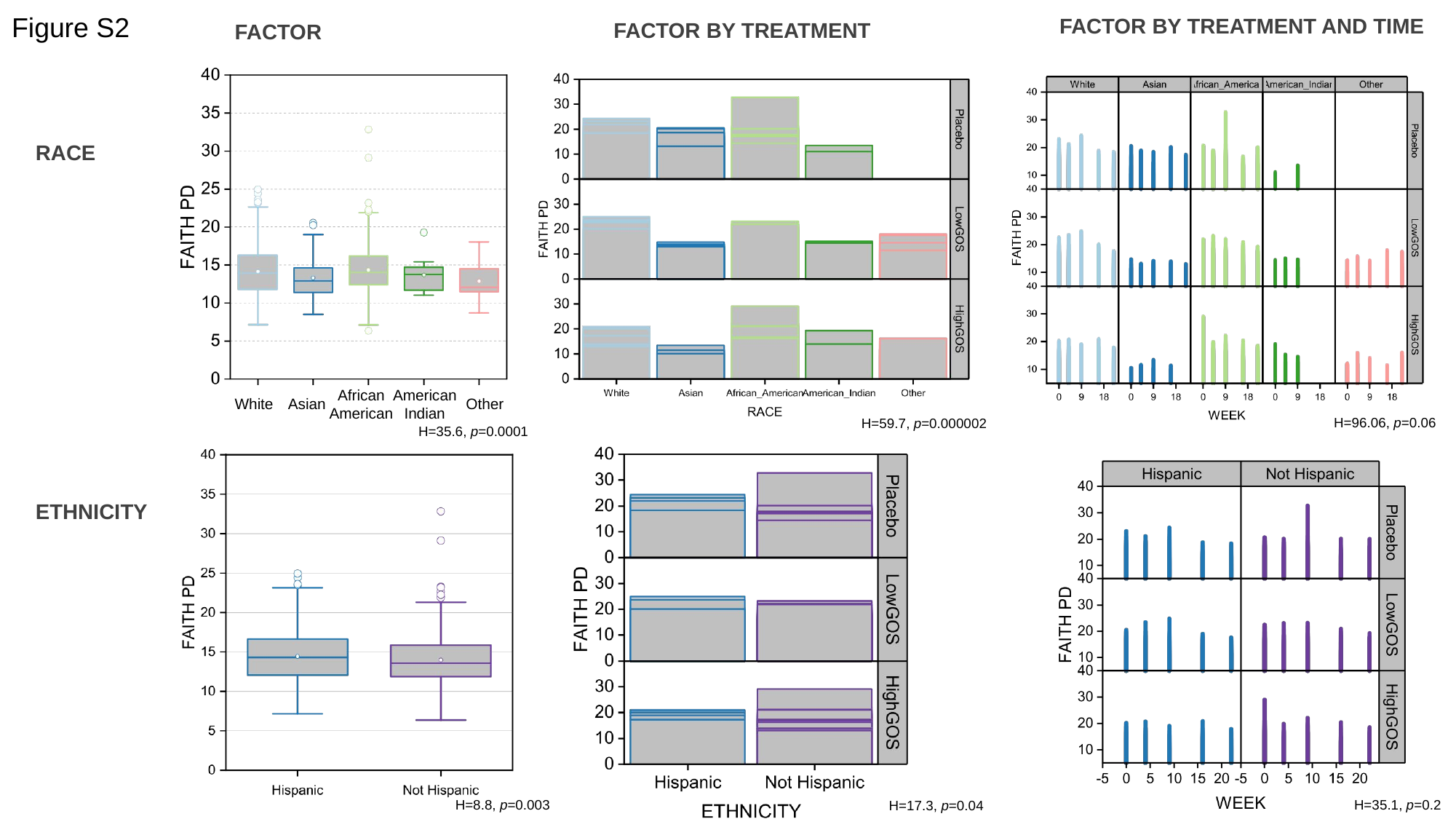

Figure S2
FACTOR BY TREATMENT AND TIME
FACTOR BY TREATMENT
FACTOR
African
American
American
Indian
White
Asian
Other
RACE
H=96.06, p=0.06
H=59.7, p=0.000002
H=35.6, p=0.0001
ETHNICITY
H=8.8, p=0.003
H=35.1, p=0.2
H=17.3, p=0.04

## Slide 5
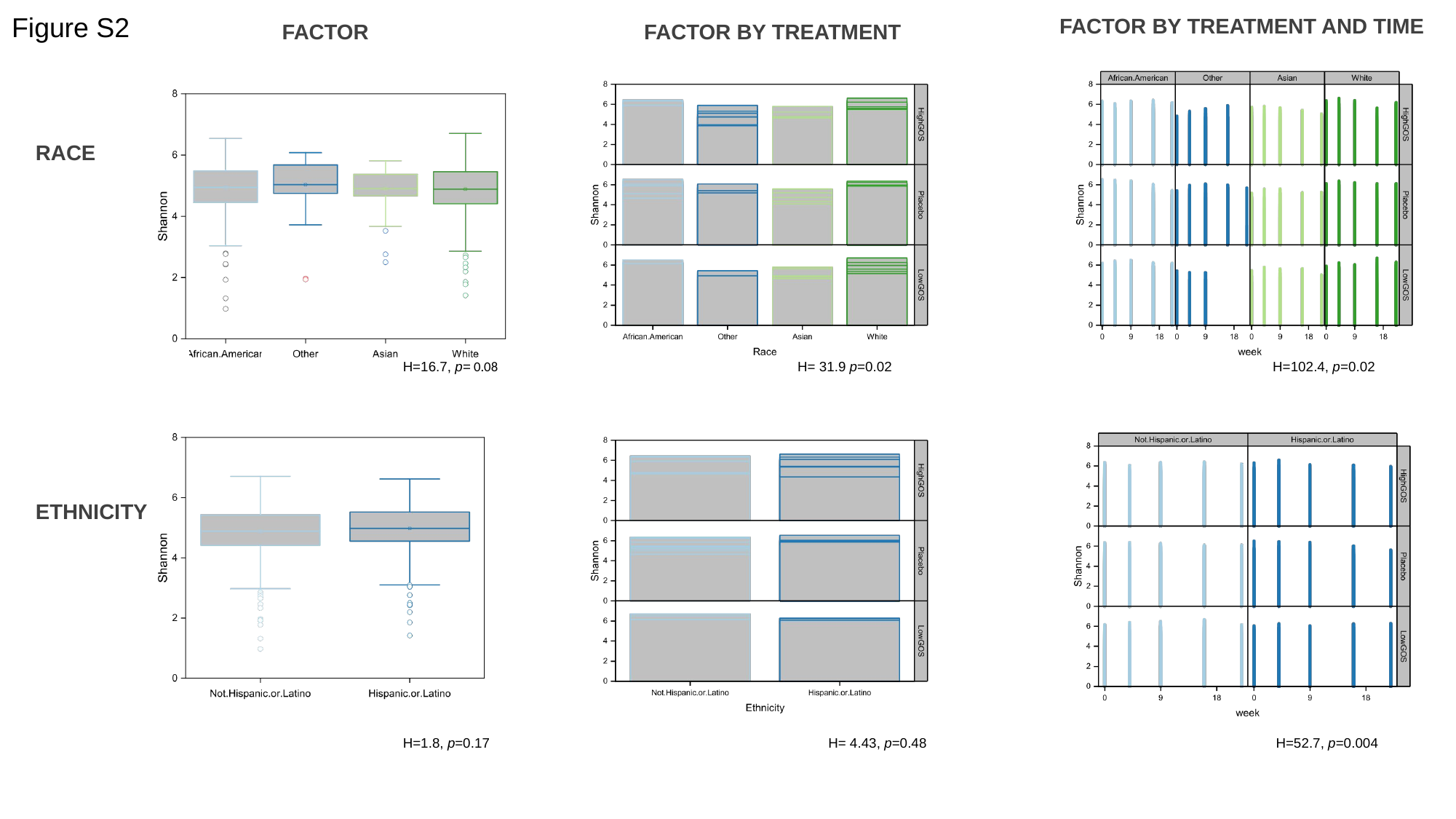

Figure S2
FACTOR BY TREATMENT AND TIME
FACTOR
FACTOR BY TREATMENT
RACE
H= 31.9 p=0.02
H=102.4, p=0.02
H=16.7, p= 0.08
ETHNICITY
H=1.8, p=0.17
H= 4.43, p=0.48
H=52.7, p=0.004

## Slide 6
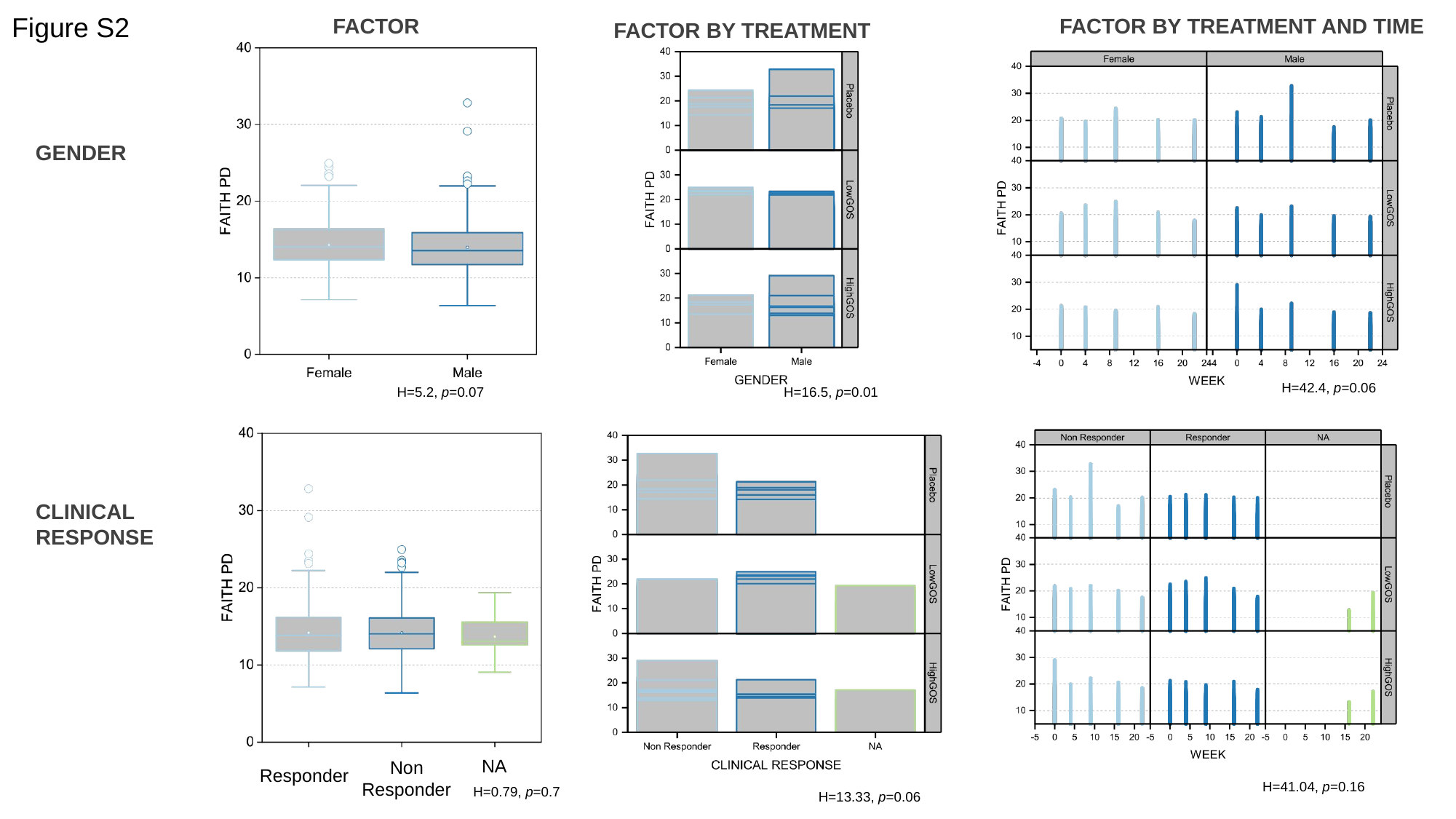

Figure S2
FACTOR
FACTOR BY TREATMENT AND TIME
FACTOR BY TREATMENT
GENDER
H=42.4, p=0.06
H=5.2, p=0.07
H=16.5, p=0.01
NA
Non
Responder
Responder
CLINICAL
RESPONSE
H=41.04, p=0.16
H=0.79, p=0.7
H=13.33, p=0.06

## Slide 7
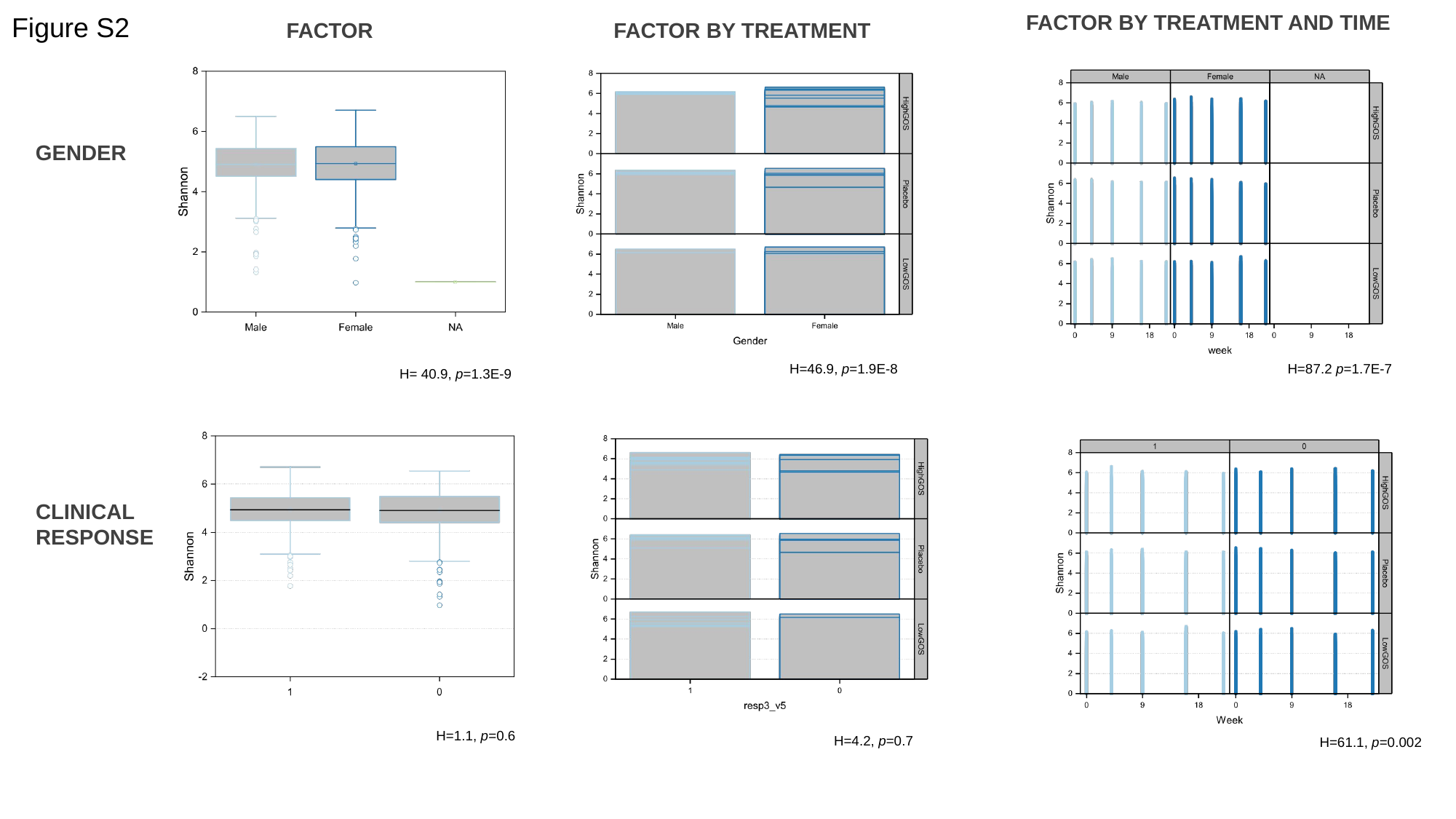

FACTOR BY TREATMENT AND TIME
Figure S2
FACTOR
FACTOR BY TREATMENT
GENDER
H=46.9, p=1.9E-8
H=87.2 p=1.7E-7
H= 40.9, p=1.3E-9
CLINICAL
RESPONSE
H=1.1, p=0.6
H=4.2, p=0.7
H=61.1, p=0.002

## Slide 8
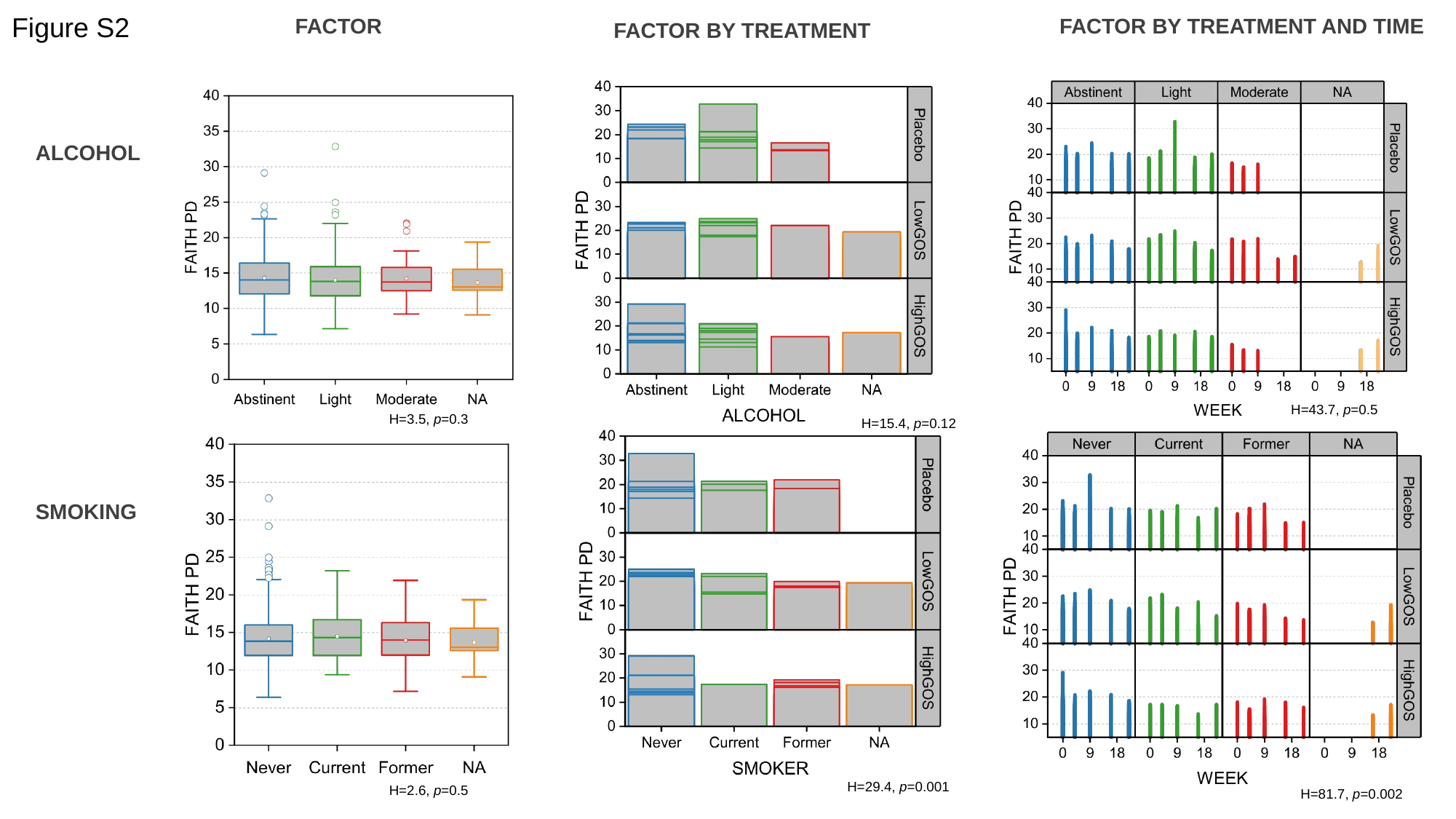

Figure S2
FACTOR
FACTOR BY TREATMENT AND TIME
FACTOR BY TREATMENT
ALCOHOL
H=43.7, p=0.5
H=3.5, p=0.3
H=15.4, p=0.12
SMOKING
H=29.4, p=0.001
H=2.6, p=0.5
H=81.7, p=0.002

## Slide 9
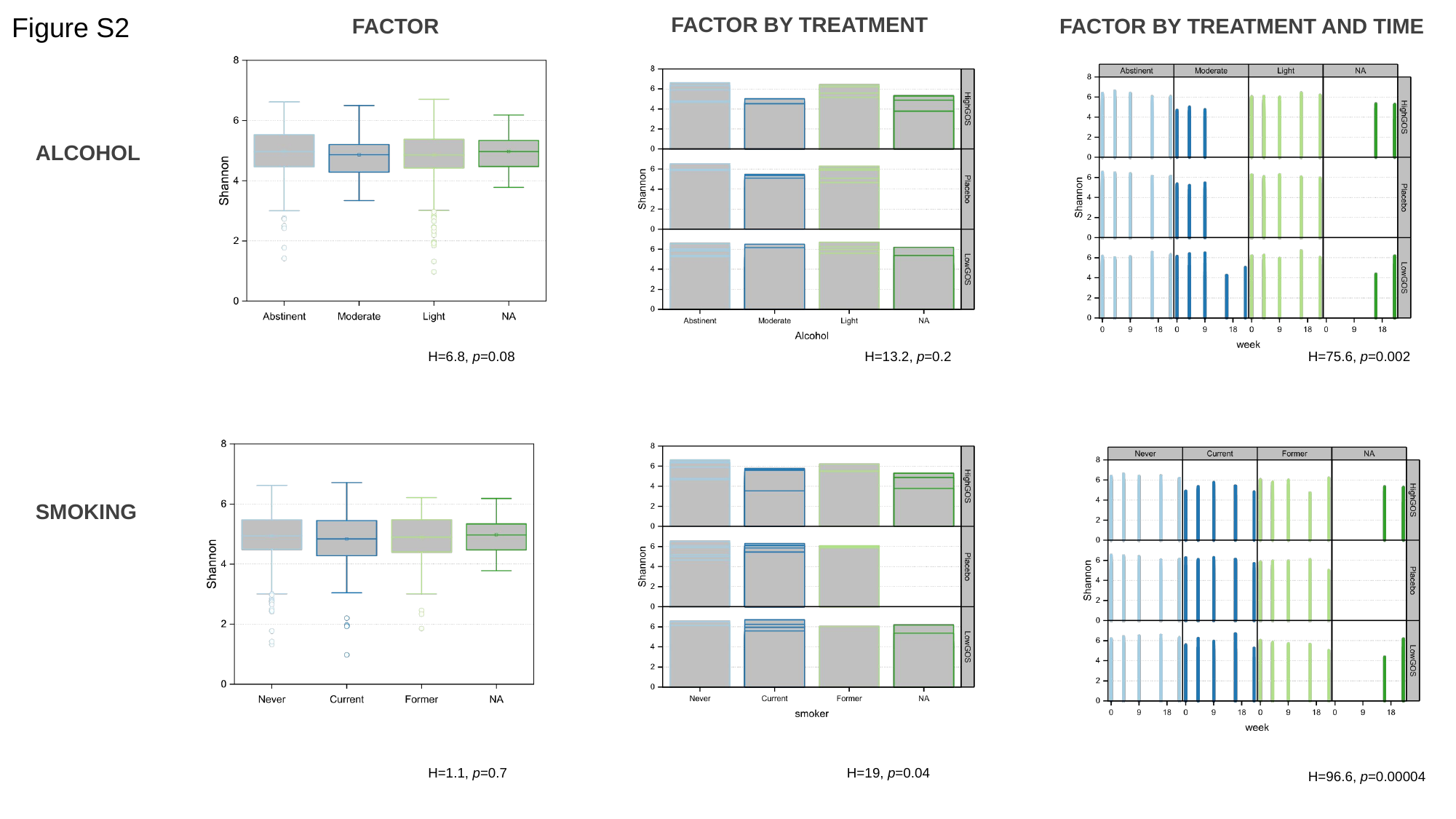

Figure S2
FACTOR BY TREATMENT
FACTOR BY TREATMENT AND TIME
FACTOR
ALCOHOL
H=13.2, p=0.2
H=75.6, p=0.002
H=6.8, p=0.08
SMOKING
H=19, p=0.04
H=1.1, p=0.7
H=96.6, p=0.00004

## Slide 10
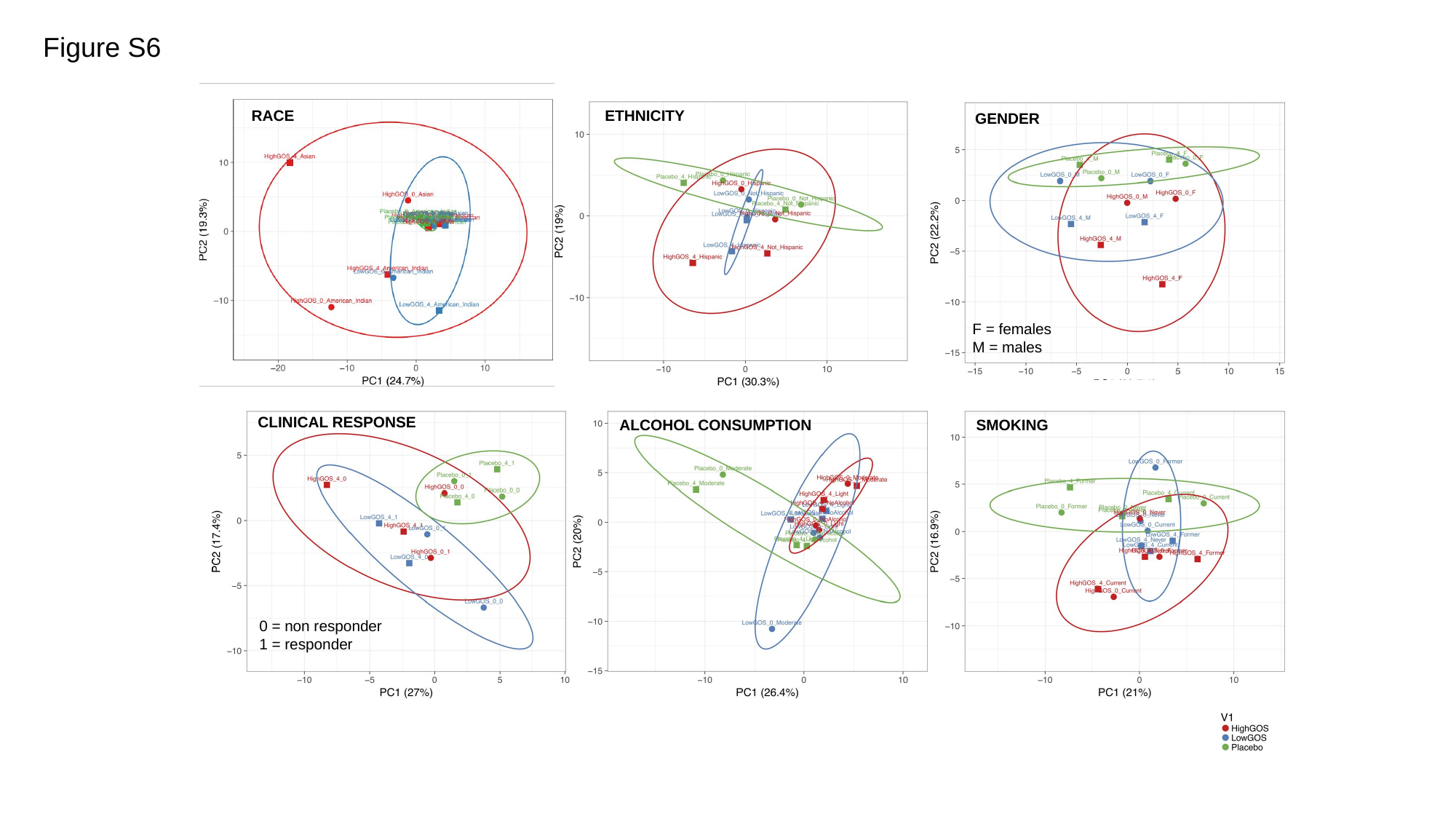

Figure S6
RACE
ETHNICITY
GENDER
F = females
M = males
CLINICAL RESPONSE
ALCOHOL CONSUMPTION
SMOKING
0 = non responder
1 = responder
