# Supplementary material for: A double-blind, 377-subject randomized study identifies Ruminococcus, Coprococcus, Christensenella, and Collinsella as long-term potential key players in the modulation of the gut microbiome of lactose intolerant individuals by galacto-oligosaccharides
Source: Gut Microbes. 2021 Aug 7;13(1):1957536. doi: 10.1080/19490976.2021.1957536 (PMC8354614; doi:10.1080/19490976.2021.1957536)
Supplement: Supplemental Material [file KGMI_A_1957536_SM7339.zip › Supplementary information/Table S1_Addendum.docx]

**Table S1.** Bifidogenic responders versus non responders in placebo and treatment groups at 31 and 61 days. Treatment groups are: 1: Placebo, 2: GOS – low dose, 3: GOS – high dose.

|  |  | 31 days | | 61 days | |
| --- | --- | --- | --- | --- | --- |
| *Targeted taxon* | ***Treatment***  ***Group*** | ***Responders (%)*** | ***Non responders (%)*** | ***Responders (%)*** | ***Non responders (%)*** |
| Actinobacteria | 1 | 54.3 | 45.7 | 59.1 | 40.9 |
|  | 2 | 68.8 | 31.2 | 52.7 | 47.3 |
|  | 3 | 73.7 | 26.3 | 56 | 44 |
| Bifidobacteriaceae_16S | 1 | 52.1 | 47.9 | 55.3 | 44.7 |
|  | 2 | 67 | 33 | 59.8 | 40.2 |
|  | 3 | 77.8 | 22.2 | 50 | 50 |
| Bifidobacterium_16S | 1 | 56.4 | 43.6 | 56.4 | 43.6 |
|  | 2 | 70.3 | 29.7 | 53.8 | 46.2 |
|  | 3 | 65.7 | 34.3 | 62.6 | 37.4 |
| B. adolescentis_16S** | 1 | 41.7 | 58.3 | 30 | 70 |
|  | 2 | 71.4 | 28.6 | 77.8 | 22.2 |
|  | 3 | 46.2 | 53.8 | 30 | 70 |
| B. adolescentis_groEL | 1 | 45.8 | 54.2 | 45.6 | 54.4 |
|  | 2 | 41.3 | 58.8 | 47.5 | 52.5 |
|  | 3 | 43.9 | 56.1 | 48.2 | 51.8 |
| B. angulatum_16S | 1 | 35.8 | 64.2 | 22.5 | 77.5 |
|  | 2 | 47.1 | 52.9 | 27.2 | 72.8 |
|  | 3 | 46.6 | 53.4 | 37.4 | 62.6 |
| B. angulatum_groEL | 1 | 42 | 58 | 50.6 | 49.4 |
|  | 2 | 69.2 | 30.8 | 63.4 | 36.6 |
|  | 3 | 58.2 | 41.8 | 48 | 52 |
| B. animalis_16S | 1 | 57.9 | 42.1 | 47.1 | 52.9 |
|  | 2 | 65.7 | 34.3 | 58.3 | 41.7 |
|  | 3 | 57.8 | 42.2 | 52.9 | 47.1 |
| B. animalis_groEL | 1 | 60.8 | 39.2 | 46.9 | 53.1 |
|  | 2 | 60.4 | 39.6 | 62.5 | 37.5 |
|  | 3 | 50 | 50 | 42.9 | 57.1 |
| B. bifidum_16S | 1 | 47.2 | 52.8 | 50.9 | 49.1 |
|  | 2 | 60.9 | 39.1 | 50 | 50 |
|  | 3 | 69.8 | 30.2 | 65.4 | 34.6 |
| B. bifidum_groEL | 1 | 48.7 | 50 | 51.3 | 48.7 |
|  | 2 | 60.7 | 39.3 | 56 | 44 |
|  | 3 | 70 | 30 | 54.4 | 45.6 |
| B. breve_16S | 1 | 69.2 | 30.8 | 37.5 | 62.5 |
|  | 2 | 63.2 | 36.8 | 63.2 | 36.8 |
|  | 3 | 66.7 | 33.3 | 41.2 | 58.8 |
| B. breve_groEL | 1 | 50.7 | 49.3 | 42.4 | 57.6 |
|  | 2 | 52.9 | 47.1 | 44.9 | 55.1 |
|  | 3 | 61.5 | 38.5 | 56.1 | 43.9 |
| B. catenulatum_16S | 1 | 51.5 | 48.5 | 52.9 | 47.1 |
|  | 2 | 61.9 | 38.1 | 56.9 | 43.1 |
|  | 3 | 73 | 27 | 63.2 | 36.8 |
| B. catenulatum_groEL** | 1 | 52.9 | 47.1 | 65 | 35 |
|  | 2 | 66.7 | 33.3 | 56.3 | 43.8 |
|  | 3 | 63.2 | 36.8 | 53.3 | 46.7 |
| B. dentium_16S | 1 | 46.1 | 52.6 | 48.6 | 51.4 |
|  | 2 | 45.7 | 54.3 | 46.4 | 53.6 |
|  | 3 | 47 | 53 | 28.8 | 71.2 |
| B. dentium_groEL | 1 | 50 | 50 | 44.9 | 55.1 |
|  | 2 | 50.6 | 49.4 | 34.2 | 65.8 |
|  | 3 | 56.6 | 43.4 | 46.9 | 53.1 |
| B. gallicum_16S** | 1 | 41.2 | 58.8 | 43.8 | 56.3 |
|  | 2 | 52.9 | 47.1 | 55.6 | 44.4 |
|  | 3 | 42.9 | 57.1 | 43.8 | 56.3 |
| B. gallicum_groEL | 1 | 44.4 | 55.6 | 49 | 51 |
|  | 2 | 45.5 | 54.5 | 47.7 | 52.3 |
|  | 3 | 51.1 | 48.9 | 42.5 | 57.5 |
| B. longum_16S | 1 | 41.5 | 41.5 | 63.8 | 36.3 |
|  | 2 | 64.6 | 35.4 | 53 | 47 |
|  | 3 | 71.9 | 28.1 | 58.8 | 41.2 |
| B. longum_groEL | 1 | 52.7 | 47.3 | 57 | 43 |
|  | 2 | 65.6 | 34.4 | 50.5 | 49.5 |
|  | 3 | 68.7 | 31.3 | 51 | 49 |

**Taxon was detected in less than 20% of samples suggesting that primers were ineffective.
