# Supplementary material for: A double-blind, 377-subject randomized study identifies Ruminococcus, Coprococcus, Christensenella, and Collinsella as long-term potential key players in the modulation of the gut microbiome of lactose intolerant individuals by galacto-oligosaccharides
Source: Gut Microbes. 2021 Aug 7;13(1):1957536. doi: 10.1080/19490976.2021.1957536 (PMC8354614; doi:10.1080/19490976.2021.1957536)
Supplement: Supplemental Material [file KGMI_A_1957536_SM7339.zip › Supplementary information/Table S2_Addendum.docx]

**Table S2.** Permutational Analysis of Variance (PERMANOVA) analysis using unweighted and weighted Unifrac distance matrices between microbiome composition and single parameters (age, gender, BMI, race, ethnicity, alcohol consumption and smoking), and between microbiome composition and treatment by factor.

|  |  | PERMANOVA UNWEIGHTED UNIFRAC | | PERMANOVA WEIGHTED UNIFRAC | |
| --- | --- | --- | --- | --- | --- |
| Single Parameters | **Number of groups (N=1332)** | **Pseudo-F** | ***P* value** | **Pseudo-F** | ***P* value** |
| Treatment1 (Placebo, GOS Low, GOS High) | 3 | 3.38988 | 0.001 | 3.16669 | 0.001 |
| Treatment2 (GOS groups combined) | 2 | 3.65196 | 0.001 | 3.96714 | 0.004 |
| Clinical response | 3 | 2.49198 | 0.001 | 5.20203 | 0.001 |
| Age | 6 | 3.67104 | 0.001 | 2.93671 | 0.001 |
| Alcohol | 4 | 2.51622 | 0.001 | 4.84889 | 0.001 |
| BMI | 6 | 3.37533 | 0.001 | 4.79159 | 0.001 |
| Ethnicity | 2 | 4.65114 | 0.001 | 7.17265 | 0.001 |
| Gender | 3 | 5.82872 | 0.001 | 9.65901 | 0.001 |
| Race | 11 | 3.32263 | 0.001 | 2.72518 | 0.001 |
| Smoking | 4 | 2.99931 | 0.001 | 4.34979 | 0.001 |
| Combined (Treatment1 by Factor) |  |  |  |  |  |
| Clinical response | 8 | 2.58713 | 0.001 | 2.73648 | 0.001 |
| Age | 16 | 3.18495 | 0.001 | 2.52713 | 0.001 |
| Alcohol | 11 | 2.52077 | 0.001 | 3.09951 | 0.001 |
| BMI | 18 | 3.20154 | 0.001 | 3.46774 | 0.001 |
| Ethnicity | 6 | 3.72061 | 0.001 | 3.93301 | 0.001 |
| Gender | 7 | 3.72503 | 0.001 | 4.76704 | 0.001 |
| Race | 19 | 2.92689 | 0.001 | 2.51686 | 0.001 |
| Smoking | 11 | 3.04796 | 0.001 | 3.29949 | 0.001 |
| Combined (Treatment2 by Factor) |  |  | 0.001 |  |  |
| Clinical response | 6 | 2.56414 | 0.001 | 3.08756 | 0.001 |
| Age | 11 | 3.33302 | 0.001 | 2.72634 | 0.001 |
| Alcohol | 8 | 2.52814 | 0.001 | 3.56113 | 0.001 |
| BMI | 12 | 3.19876 | 0.001 | 3.85386 | 0.001 |
| Ethnicity | 4 | 4.29481 | 0.001 | 4.28994 | 0.001 |
| Gender | 5 | 4.2005 | 0.001 | 6.40222 | 0.001 |
| Race | 15 | 3.16422 | 0.001 | 2.59795 | 0.001 |
| Smoking | 8 | 3.2504 | 0.001 | 3.37828 | 0.001 |
| Treatments by Time (Visit week) |  |  |  |  |  |
| Treatment1 | 15 | 1.98295 | 0.001 | 3.86627 | 0.001 |
| Treatment2 | 10 | 2.37039 | 0.001 | 5.33277 | 0.001 |
| Combined (Treatment1 X Time X Factor) |  |  |  |  |  |
| Clinical response | 34 | 1.59259 | 0.001 | 2.46478 | 0.001 |
| Age | 72 | 1.52182 | 0.001 | 1.85347 | 0.001 |
| Alcohol | 45 | 1.49259 |  | 2.38071 | 0.001 |
| BMI |  |  |  |  |  |
| Ethnicity | 30 | 1.75018 | 0.001 | 2.96801 | 0.001 |
| Gender | 31 | 1.83046 | 0.001 | 3.01425 | 0.001 |
| Race |  |  |  |  |  |
| Smoking | 49 | 1.61393 | 0.001 | 2.21897 | 0.001 |
| Combined (Treatment2 X Time X Factor) |  |  |  |  |  |
| Clinical response | 23 | 1.81606 | 0.001 | 3.13542 | 0.001 |
| Age | 49 | 1.67341 | 0.001 | 2.16098 | 0.001 |
| Alcohol | 31 | 1.64326 | 0.001 | 2.82404 | 0.001 |
| BMI | 55 | 1.60043 | 0.001 | 2.6682 | 0.001 |
| Ethnicity | 20 | 2.05149 | 0.001 | 3.8305 | 0.001 |
| Gender | 21 | 2.0875 | 0.001 | 4.07051 | 0.001 |
| Race |  |  |  |  |  |
| Smoking | 33 | 1.81874 | 0.001 | 2.56124 | 0.001 |
