# Supplementary material for: A double-blind, 377-subject randomized study identifies Ruminococcus, Coprococcus, Christensenella, and Collinsella as long-term potential key players in the modulation of the gut microbiome of lactose intolerant individuals by galacto-oligosaccharides
Source: Gut Microbes. 2021 Aug 7;13(1):1957536. doi: 10.1080/19490976.2021.1957536 (PMC8354614; doi:10.1080/19490976.2021.1957536)
Supplement: Supplemental Material [file KGMI_A_1957536_SM7339.zip › Supplementary information/Table S3_Addendum.docx]

| **Table S3.** High-throughput qPCR primers targeting bacterial groups and species within the *Lactobacillus* genus using the Access Array AA 24.192 (Fluidigm Corporation, San Francisco, CA, USA).   \| *Target Species* \|  \| *Primer Name* \| *Sequence 5' to 3'* \| *Target Gene* \| *Product Size* \| *Reference* \| \| --- \| --- \| --- \| --- \| --- \| --- \| --- \| \| *16S Universal (1)* \| Fwd \| Uni_V4_F \| CAGCAGCCGCGGTAATAC \| 16S \| 389 \| [1] \| \| Rev \| Uni_V4_R \| CCGTCAATTTCTTTGAGTTT \| \| *16S Universal (2)* \| Fwd \| Uni_F \| GTGSTGCAYGGYYGTCGTCA \| 16S \| 194 \| [2] \| \| Rev \| Uni_R \| ACGTCRTCCMCNCCTTCCTC \| \| *16S Universal (3)* \| Fwd \| 8F \| AGAGTTTGATCCTGGCTCAG \| 16S \| 330 \| [3-5] \| \| Rev \| 8F_B \| AGGGTTCGATTCTGGCTCAG \| \| Rev \| Uni_338_R \| GCTGCCTCCCGTAGGAGT \| \| *Firmicutes* \| Fwd \| Firmicutes_F \| GGAGYATGTGGTTTAATTCGAAGCA \| 16S \| 126 \| [6] \| \| Rev \| Firmicutes_R \| AGCTGACGACAACCATGCAC \| \| *Total Lactobacillus* \| Fwd \| Lactobacillus_F \| AGCAGTAGGGAATCTTCCA \| 16S \| 341 \| [7] \| \| Rev \| Lactobacillus_R \| CACCGCTACACATGGAG \| \| *L. delbruecki* \| Fwd \| L_delb_F \| GGRTGATTTGTTGGACGCTAG \| 16S \| 138 \| [8] \| \| Rev \| L_delb_R \| GCCGCCTTTCAAACTTGAATC \| \| *L. gallinarum* \| Fwd \| L_gallinarum_F \| TCAGGACCTTGTACTACCTTGTAA \| 16S \| 180 \| [9] \| \| Rev \| L_gallinarum_R \| TGCTACTAAGGCTGAAATCGT \| \| *L. intestinalis* \| Fwd \| L_intestin_F \| GGTGATGACGCTGGGAAC \| 16S \| 130 \| [10] \| \| Rev \| L_intestin_R \| AAGCAATAGCCATGCAGC \| \| *L. acidophilus Group* \| Fwd \| L_acidGroup1_F \| AGCGAGCGGAACTAACAGATTTAC \| 16S \| 154 \| [11] \| \| Rev \| L_acidGroup1_R \| AGCTGATCATGCGATCTGCTT \| \| *L. murinus* \| Fwd \| L_murinus_F \| GAACGAAACTTCTTTATCACC \| 16S \| 146 \| [10] \| \| Rev \| L_murinus_R \| TAGCATAGCCACCTTTTACA \| \| *L. acidophilus Group* \| Fwd \| L_acidGroup2_F \| GCGAGCGGAACTAACAGATTT \| 16S \| 150 \| [12] \| \| Rev \| L_acidGroup2_R \| TGATCATGCGATCTGCTTTCT \| \| *L. casei Group* \| Fwd \| L_caseiGroup_F \| ACCGCATGGTTCTTGGC \| 16S \| 296 \| [13] \| \| Rev \| L_caseiGroup_R \| CCGACAACAGTTACTCTGCC \| \| *L. reuteri* \| Fwd \| L_reuteri_F_IS \| CAGACAATCTTTGATTGTTTAG \| IS \| 305 \| [14] \| \| Rev \| L_reuteri_R_IS \| GCTTGTTGGTTTGGGCTCTTC \| \| *L. plantarum* \| Fwd \| L_plantarum_F \| CTCTGGTATTGATTGGTGCTTGCAT \| 16S \| 54 \| [13] \| \| Rev \| L_plantarum_R \| GTTCGCCACTCACTCAAATGTAAA \| \| *L. fermentum* \| Fwd \| L_fermentum_F \| GCACCTGATTGATTTTGGTCG \| 16S \| 103 \| [15] \| \| Rev \| L_fermentum_R \| GGTATTAGCATCTGTTTCCAAATG \| \| *L. salivarius* \| Fwd \| L_salivarius_F \| GATCGCATGATCCTTAGATGAA \| 16S \| 130 \| [16] \| \| Rev \| L_salivarius_R \| GCCGATCAACCTCTCAGTTC \|   **References** |
| --- | --- | --- | --- | --- | --- | --- | --- | --- | --- | --- | --- | --- | --- | --- | --- | --- | --- | --- | --- | --- | --- | --- | --- | --- | --- | --- | --- | --- | --- | --- | --- | --- | --- | --- | --- | --- | --- | --- | --- | --- | --- | --- | --- | --- | --- | --- | --- | --- | --- | --- | --- | --- | --- | --- | --- | --- | --- | --- | --- | --- | --- | --- | --- | --- | --- | --- | --- | --- | --- | --- | --- | --- | --- | --- | --- | --- | --- | --- | --- | --- | --- | --- | --- | --- | --- | --- | --- | --- | --- | --- | --- | --- | --- | --- | --- | --- | --- | --- | --- | --- | --- | --- | --- | --- | --- | --- | --- | --- | --- | --- | --- | --- | --- | --- | --- | --- | --- | --- | --- | --- | --- | --- | --- | --- | --- | --- | --- | --- | --- | --- | --- | --- | --- | --- | --- | --- | --- | --- | --- | --- | --- | --- | --- | --- | --- | --- | --- | --- | --- | --- | --- | --- | --- | --- | --- | --- | --- | --- | --- | --- | --- | --- | --- | --- | --- | --- | --- | --- | --- | --- |

1. Hermann-Bank ML, Skovgaard K, Stockmarr A, Larsen N, Molbak L: **The Gut Microbiotassay: a high-throughput qPCR approach combinable with next generation sequencing to study gut microbial diversity**. *BMC Genomics* 2013, **14**:788.

2. Maeda H, Fujimoto C, Haruki Y, Maeda T, Kokeguchi S, Petelin M, Arai H, Tanimoto I, Nishimura F, Takashiba S: **Quantitative real-time PCR using TaqMan and SYBR Green for Actinobacillus actinomycetemcomitans, Porphyromonas gingivalis, Prevotella intermedia, tetQ gene and total bacteria**. *FEMS Immunol Med Microbiol* 2003, **39**(1):81-86.

3. Edwards U, Rogall T, Blocker H, Emde M, Bottger EC: **Isolation and direct complete nucleotide determination of entire genes. Characterization of a gene coding for 16S ribosomal RNA**. *Nucleic Acids Res* 1989, **17**(19):7843-7853.

4. Fierer N, Hamady M, Lauber CL, Knight R: **The influence of sex, handedness, and washing on the diversity of hand surface bacteria**. *Proc Natl Acad Sci U S A* 2008, **105**(46):17994-17999.

5. Martinez I, Kim J, Duffy PR, Schlegel VL, Walter J: **Resistant starches types 2 and 4 have differential effects on the composition of the fecal microbiota in human subjects**. *PLoS One* 2010, **5**(11):e15046.

6. Guo X, Xia X, Tang R, Zhou J, Zhao H, Wang K: **Development of a real-time PCR method for Firmicutes and Bacteroidetes in faeces and its application to quantify intestinal population of obese and lean pigs**. *Lett Appl Microbiol* 2008, **47**(5):367-373.

7. Rinttila T, Kassinen A, Malinen E, Krogius L, Palva A: **Development of an extensive set of 16S rDNA-targeted primers for quantification of pathogenic and indigenous bacteria in faecal samples by real-time PCR**. *J Appl Microbiol* 2004, **97**(6):1166-1177.

8. Zhang R, Daroczy K, Xiao B, Yu L, Chen R, Liao Q: **Qualitative and semiquantitative analysis of Lactobacillus species in the vaginas of healthy fertile and postmenopausal Chinese women**. *J Med Microbiol* 2012, **61**(Pt 5):729-739.

9. Moser A, Wuthrich D, Bruggmann R, Eugster-Meier E, Meile L, Irmler S: **Amplicon Sequencing of the slpH Locus Permits Culture-Independent Strain Typing of Lactobacillus helveticus in Dairy Products**. *Front Microbiol* 2017, **8**:1380.

10. Gomes-Neto JC, Mantz S, Held K, Sinha R, Segura Munoz RR, Schmaltz R, Benson AK, Walter J, Ramer-Tait AE: **A real-time PCR assay for accurate quantification of the individual members of the Altered Schaedler Flora microbiota in gnotobiotic mice**. *J Microbiol Methods* 2017, **135**:52-62.

11. Byun R, Nadkarni MA, Chhour KL, Martin FE, Jacques NA, Hunter N: **Quantitative analysis of diverse Lactobacillus species present in advanced dental caries**. *J Clin Microbiol* 2004, **42**(7):3128-3136.

12. Torok VA, Hughes RJ, Mikkelsen LL, Perez-Maldonado R, Balding K, MacAlpine R, Percy NJ, Ophel-Keller K: **Identification and characterization of potential performance-related gut microbiotas in broiler chickens across various feeding trials**. *Appl Environ Microbiol* 2011, **77**(17):5868-5878.

13. Matsuda K, Tsuji H, Asahara T, Matsumoto K, Takada T, Nomoto K: **Establishment of an analytical system for the human fecal microbiota, based on reverse transcription-quantitative PCR targeting of multicopy rRNA molecules**. *Appl Environ Microbiol* 2009, **75**(7):1961-1969.

14. Haarman M, Knol J: **Quantitative real-time PCR analysis of fecal Lactobacillus species in infants receiving a prebiotic infant formula**. *Appl Environ Microbiol* 2006, **72**(4):2359-2365.

15. Schwendimann L, Kauf P, Fieseler L, Gantenbein-Demarchi C, Miescher Schwenninger S: **Development of a quantitative PCR assay for rapid detection of Lactobacillus plantarum and Lactobacillus fermentum in cocoa bean fermentation**. *J Microbiol Methods* 2015, **115**:94-99.

16. Saint-Cyr MJ, Haddad N, Taminiau B, Poezevara T, Quesne S, Amelot M, Daube G, Chemaly M, Dousset X, Guyard-Nicodeme M: **Use of the potential probiotic strain Lactobacillus salivarius SMXD51 to control Campylobacter jejuni in broilers**. *Int J Food Microbiol* 2017, **247**:9-17.
